# Fructose dehydration to 5-hydroxymethylfurfural over supported acid catalysts: impact of acid chemical species

**DOI:** 10.1039/d6se00627b

**Published:** 2026-08-05

**Authors:** Aina Syahida Jamal, Cameron-Alexander H. Price, Molly Smith, Daniel Lee, Jesús Esteban, Christopher M. A. Parlett

**Affiliations:** a Department of Chemical Engineering, The University of Manchester Oxford Road Manchester M13 9PL UK christopher.parlett@manchester.ac.uk; b UK Catalysis Hub, Research Complex at Harwell, Rutherford Appleton Laboratory Harwell Oxfordshire OX11 0FA UK; c Department of Chemical Engineering and Materials, Faculty of Chemical Sciences, Complutense University of Madrid 28040 Madrid Spain; d University of Manchester at Harwell, Diamond Light Source, Harwell Science and Innovation Campus Didcot Oxfordshire OX11 0DE UK

## Abstract

Biomass-derived 5-hydroxymethylfurfural (HMF) represents a crucial platform molecule in the advancement of sustainable chemical production, being produced *via* acid-catalysed dehydration of sugars. This study investigates the impact of three supported organic acids, namely sulphonic (RSO_3_H/stellate), phosphonic (RPO_3_H_2_/stellate), and carboxylic (RCOOH/stellate) acids, grafted on mesoporous silica nanospheres. Deployment of these as catalytic species facilitates the evaluation of acid strength on HMF dehydration and the impact on subsequent undesirable side reactions, including polyfuranic humin formation. While increased acid strength promotes higher fructose conversion, the weaker carboxylic acid catalysts consistently exhibit superior long-term selectivity towards HMF. Maintaining high HMF selectivity is reliant on preventing further reactions of it, with acid-catalysed aldol condensations yielding humins more favourably over stronger acid sites. After 45 min at 165 °C, HMF selectivity is greater for RCOOH/stellate (85% at 53% conversion) relative to RSO_3_H/stellate (78% at 83% conversion) and RPO_3_H_2_/stellate (71% at 91% conversion). As reaction temperature escalates, a trade-off between elevated conversion and diminishing selectivity is apparent for all catalytic materials. At 185 °C, conversion reaches 70% in only 5 min with HMF selectivity of 89%, but at the expense of reduced long-term process selectivity (HMF selectivity ∼23% at 45 min). The catalyst's green credentials are further apparent from a facile reactivation protocol, which restores >85% of the initial catalyst activity.

## Abstract 2

The acid strength of grafted catalytic sites on porous silica nanospheres impacts the rate and selectivity of fructose dehydration to HMF; however, reaction parameters (temperature and time) also prove to be critical.

## Graphical abstract

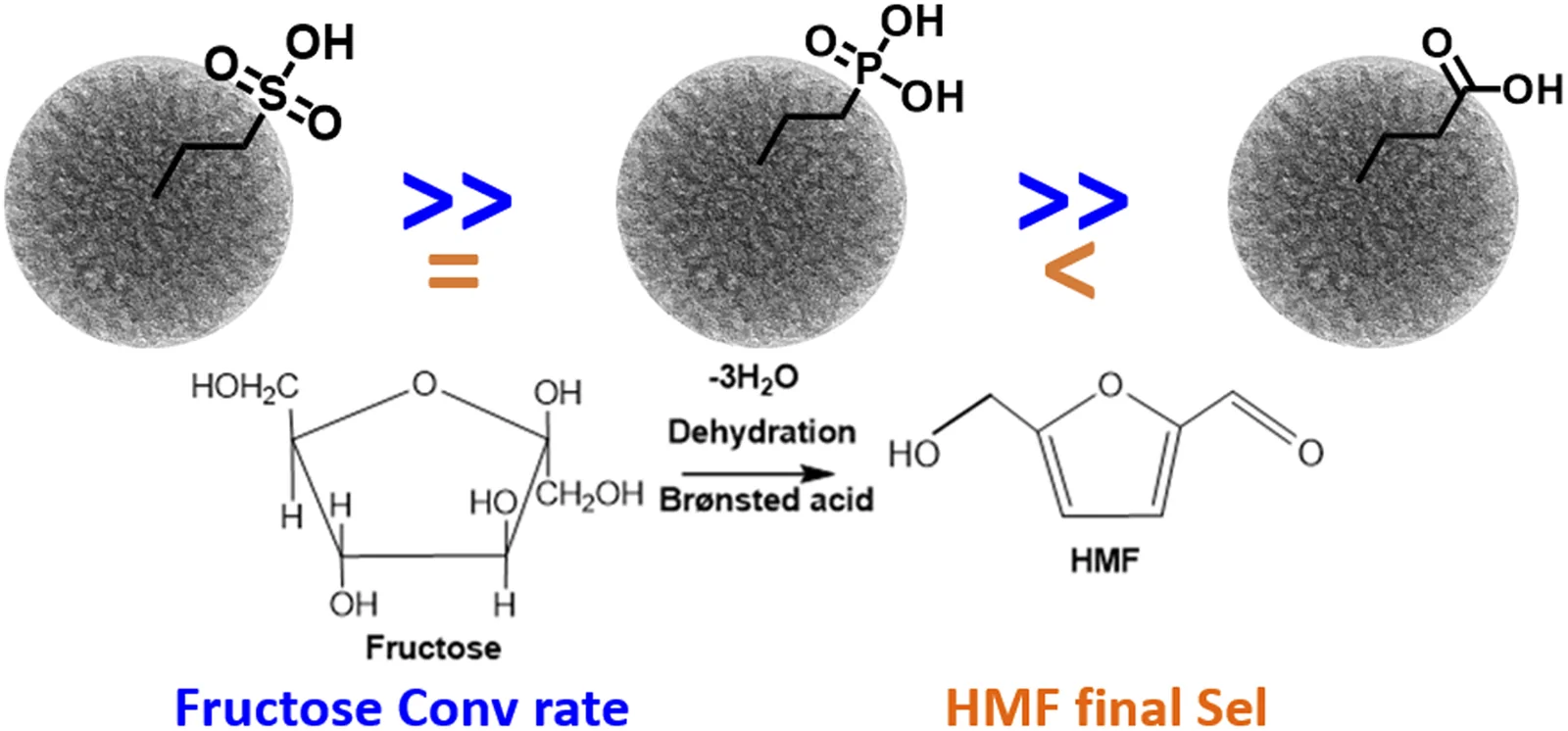

## Introduction

The development of catalytic technologies for the efficient transformation of underutilised renewable feedstocks into useful fuels and chemicals is paramount in the drive to a sustainable chemical industry. These advancements will facilitate the embracing of the circular economy concept by the transition away from the current linear production model.^1,2^ Biomass, specifically second generation non-edible lignocellulose, is a key resource to this step-change due to its wide availability,^3^ which, through selective transformations, can either complement or replace traditional petrochemical-based chemicals and chemical building blocks within the sector without impacting the global capacity to feed humanity.^4^

**Scheme 1:**
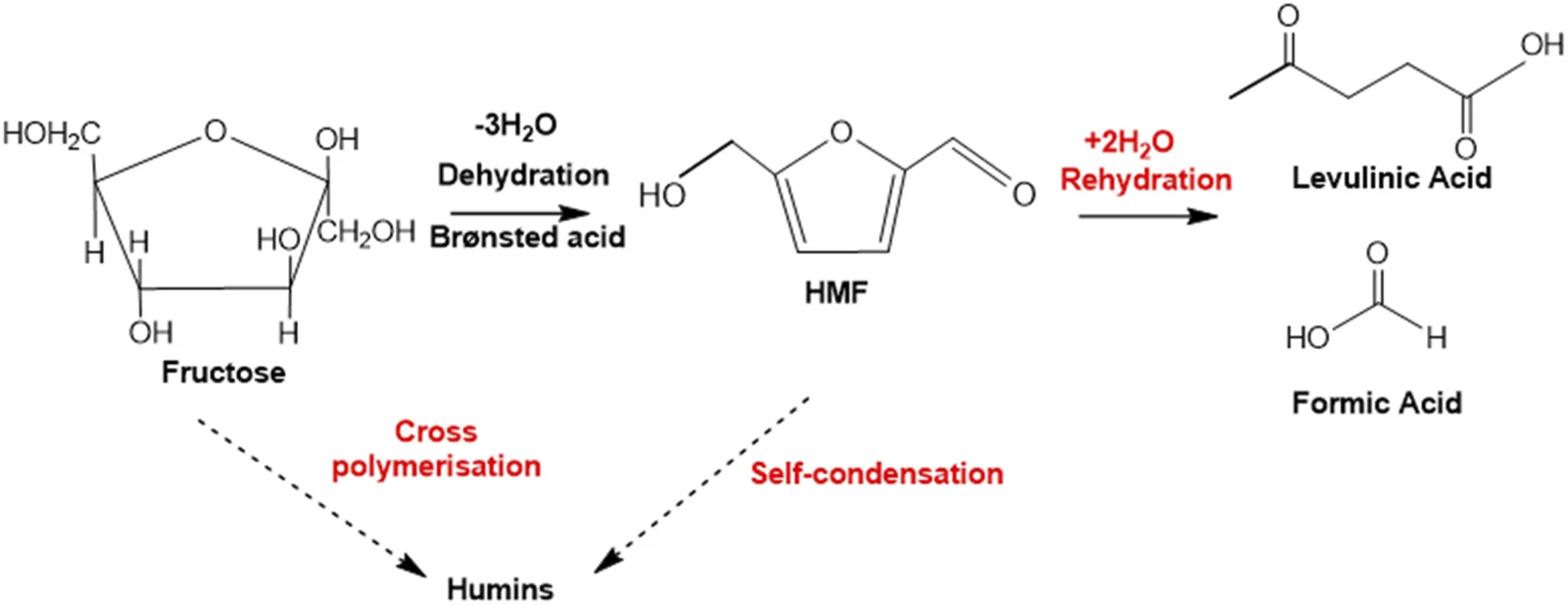
HMF production pathway from fructose through dehydration alongside undesired side reactions.

5-Hydroxymethylfurfural (HMF), a key platform chemical in the emerging bioeconomy, is known for its versatility, being an intermediate in the synthesis of fine chemicals, fuels and polymer monomers *via* an array of different chemical transformations.^4,5^ For example, it can be selectively oxidised to either 5-hydroxymethyl-2-furancarboxylic acid, a compound of interest within pharmaceutical applications,^6,7^ or further oxidised to 2,5-furandicarboxylic acid, a monomer of polyethylene furanoate, a bio-based alternative to petroleum-derived polyethylene terephthalate.^8,9^ While selective hydrogenation of HMF can yield 2,5-dimethylfuran a promising alternative biofuel to bioethanol.^10^ HMF is produced by acid-catalysed dehydration of fructose (Scheme 1),^11^ with fructose obtained *via* cellulose saccharification and subsequent glucose isomerisation. The dehydration step, catalysed by Brønsted acids, involves the loss of three water molecules, which, due to substrate solubility, is commonly conducted using water as the solvent.^12^ However, side reactions reduce HMF yields, with levulinic and formic acid arising from rehydration, whereas self or cross (with residual sugars and *in situ* formed ring-opened intermediate 2,5-dioxo-6-hydroxyhexanal)^13^ aldol condensations lead to polymeric humins.^14,15^

Acid catalysts investigated for fructose dehydration span homogeneous and heterogeneous species,^16^ and while HCl^17^ and H_2_SO_4_ ^14^ are viable, concerns over corrosion and separation hinder their uptake. Homogeneous Brønsted acids have been studied for fructose conversion to HMF, with Körner *et al.*^18^ showing that phosphoric, sulphuric, and acetic acids can achieve similar HMF yields under suitably acidic conditions and reaction lengths. For example, phosphoric acid at pH 1 affords ∼45% HMF yield in 13 minutes (∼87% conversion), while sulphuric acid at pH 1.1 attained 40% HMF yield in 32 minutes (∼83% fructose conversion). At pH 2, acetic acid reaches ∼48% HMF yield after 243 minutes (∼95% conversion). Homogeneous *para*-toluenesulfonic acid (*p*-TSA) has likewise been applied for fructose dehydration, as reported by Sajid *et al.*^19^ They demonstrated that solvent was key, with maximum HMF yields attained with DMSO (∼79%), whereas water resulted in a yield of only ∼18%. However, it is known that DMSO alone promotes high HMF yields from fructose dehydration.^20^ Thus, development of heterogeneous systems have been the focus of significant efforts,^21^ with supported sulphonic acid species strong candidates due to strong Brønsted acidity and versatility. The latter, in part, is achievable *via* the tunability of commonly employed silica based support architectures,^22^ which impart beneficial characteristics, including chemical inertness,^23^ stability, and high surface areas,^24^ whilst pore sizes can span the micro, meso and macropore domains.^25^ Furthermore, ordered mesoporous architecture can be tailored with 2-dimensional materials, including MCM-41 and SBA-15,^26,27^ as well as 3-dimensional frameworks such as SBA-16 and KIT-6,^28^ with the latter proving ideal due to their facilitating internal mass transport.^29^ Architectures can be further developed to span two pore size domains, with hierarchical mesoporous SBA-15 and KIT-6 reported, with both being beneficial for the conversion of bulky substrates.^30,31^ In heterogeneous catalytic systems, sulphonic acids are widely grafted onto porous inorganic supports such as MCM-41, zeolites, and SBA-15 to combine strong Brønsted acidity with high surface area, controlled pore architectures and improved hydrothermal stability for HMF production. While sulphonic acid grafted on MCM-41 has shown high fructose conversions (∼90%) with good HMF selectivity (∼77%) in water/MIBK system,^32^ improved performance was reported for a weaker acid species resulting from sulphuric acid impregnation of MCM-14 and calcination. Hierarchical zeolites have also been used as supports, making use of the inherent acid sites alongside the additional acidity from the grafted sulphonic species to achieve HMF selectivity of ∼73% with fructose conversion of ∼88%.^33^ Synergistic effects from combining sulphonic acidity with sulfoxide species on SBA-15 result in high HMF selectivity 79% (at 87% fructose conversion),^34^ with the sulfoxide groups mimicking DMSO in stabilising intermediates; DMSO being a commonly applied solvent which can achieve high yields in the absence of a catalyst.^20^ Thus, whilst sulphonic acids have been successfully immobilised on different support materials, their best performance is achieved either in biphasic systems or in the presence of a co-solvent. Consistent with the review by Jiang *et al.*^35^ which illustrates that chemical transformations of HMF into value-added products are strongly governed by the catalytic system and catalyst design. So whilst sulfonic acid species have received significant interest, supported carboxylic and phosphonic acid groups are less well investigated. As with sulphonic acids,^36^ periodic mesoporous organosilicas have been employed for supporting carboxylic acidity, demonstrating that the milder acidity can drive fructose dehydration (albeit in DMSO),^37^ while homogeneous equivalents further illustrate the potential of other acidic species for this chemistry.^16^

Among the diversity presented by mesoporous silicas, porous nanospheres, with unique internal architectures, offer promising and exciting alternatives to the more widely studied materials of the MCM and SBA families.^38^ The reduced size of the nanosphere morphology diminishes the maximum in-pore diffusion distance, allowing for enhanced HMF selectivity, the latter by reducing the potential subsequent side reactions by minimising residence times of products within the pore network, *i.e.*, reducing encounters of HMF with the acid catalytic sites which facilitates unwanted humin formation.^15^ Similarly, these have proven beneficial for intracellular drug delivery *via* rapid diffusion of bulky molecules.^39^ Thus, optimising performance by improving mass diffusion and active site exposure is a key area of investigation.^22^ Core–shell structures can address both aspects, representing another class of silica spheres. For example, the incorporation of a polyionic liquid core within a silica shell allows for uniform distribution of active sites, which results in high HMF yields (∼77%) from glucose after 0.5 h at 70 °C, albeit in DMSO.^40^ An alternative is to introduce a magnetic core to aid catalyst isolation,^41^ which would prove beneficial in reaction systems where solid wastes are generated. Further increases in the suitability of catalytic systems include energy-efficient heating, *via* microwave irradiation, obtaining high HMF yields within short reaction times.^42,43^

Here, the deployment of different acid functionalities on mesoporous stellate silica nanospheres is reported, aiming to leverage the distinctive architecture of the support for the efficient dehydration of fructose to HMF. By anchoring diverse acid functionalities (sulphonic, carboxylic, and phosphonic), the potential impact of acid type on catalyst performance will be elucidated. Specifically, contributing to assessing how differences in acid strength influence both conversion and selectivity, including its role in undesirable humin formation.

## Experimental section

### Materials and methods

d-(−)-Fructose (biochemistry grade), d-(+)-glucose (≥99.5%), levulinic acid (98%), formic acid (HPLC grade), furfural (99%, ACS reagent), (3-mercaptopropyl)triethoxysilane (≥80%, GC technical grade), HMF (≥99%), hexadecyltrimethylammonium *p*-toluenesulfonate (≥98%), and potassium chloride (≥99%) were purchased from Sigma-Aldrich. Tetraethyl orthosilicate (≥99%) and hydrochloric acid (37%) were purchased from Thermo Fischer Scientific. Hydrogen peroxide solution (30 wt% in water) was purchased from Merck Life Science UK. Triethoxysilylbutyraldehyde (90%) and diethylphosphatoethyltriethoxysilane (95%) were purchased from Fluorochem Limited. Triethanolamine (98%, reagent grade) was purchased from VWR International. 1-(2-Furyl)-2-hydroxyethanone (95%) was purchased from Activate Scientific UK. All chemicals were used as received without further purification. Deionised water with a resistivity of 18.2 mΩ cm was provided from a Millipore Milli-Direct Q purification system.

### Catalyst synthesis

#### Stellate silica support synthesis

Stellate silica was prepared by the method of Zhang *et al.*^44^ Hexadecyltrimethylammonium *p*-toluenesulfonate (9.1 g), triethanolamine (1.3 g), and water (479 cm^3^) were stirred at 1200 rpm for 1 h at 80 °C. Tetraethyl orthosilicate (78.2 cm^3^) was added and the solution was left to react for 2 h. The solid silica product was isolated by centrifugation (8000 rpm, 0.5 h) on a Sigma 3-16KL centrifuge with a 12 310-biosafe rotor and washed with deionised water (479 cm^3^) in triplicate before being dried at room temperature overnight. The organic mesopore template was removed by calcination at 550 °C (ramp rate 1 °C min^−1^) for 5.5 h under static air.

#### Phosphonic acid functionalisation

Stellate silica (5 g) was refluxed in ethanol (150 cm^3^) under stirring at 900 rpm. Diethylphosphatotriethoxysilane (7.5 cm^3^) was added and the solution was refluxed for 24 h. The solid was isolated by centrifugation (8000 rpm, 0.5 h), washed with ethanol (150 cm^3^) in triplicate and dried overnight at room temperature. The dried solid was refluxed in concentrated hydrochloric acid (37%, 108 cm^3^) for 24 h, before isolation by centrifugation (8000 rpm, 0.5 h) and washing with deionised water to pH 7. The solid was dried overnight at room temperature prior to further drying at 80 °C for 24 h. The final catalyst was denoted as RPO_3_H_2_/stellate.

#### Sulphonic acid functionalisation

Stellate silica (5 g) in ethanol (150 cm^3^) was refluxed under stirring at 900 rpm. (3-Mercaptopropyl)triethoxysilane (5 cm^3^) was added and the solution was refluxed for 24 h. The solid was isolated by centrifugation (8000 rpm, 0.5 h), washed in triplicate with ethanol (150 cm^3^) and dried overnight at room temperature. The solid was stirred in an aqueous hydrogen peroxide solution (150 cm^3^) for 24 h at room temperature, before isolation by centrifugation (8000 rpm, 0.5 h). The solid was washed with deionised water in triplicate and dried overnight at room temperature prior to further drying at 80 °C for 24 h. The resultant catalyst was denoted as RSO_3_H/stellate.

#### Carboxylic acid functionalisation

Stellate silica (5 g) in ethanol (150 cm^3^) was refluxed under stirring at 900 rpm. Triethoxysilylbutyraldehyde (5 cm^3^) was added and the solution was refluxed for 24 h. The solid was isolated by centrifugation (8000 rpm, 0.5 h), washed with ethanol (150 cm^3^) in triplicate and dried overnight at room temperature. The solid was stirred in an aqueous hydrogen peroxide solution (150 cm^3^) for 24 h at room temperature. The solid was isolated by centrifugation (8000 rpm, 0.5 h), washed with deionised water in triplicate and dried overnight at room temperature prior to further drying at 80 °C for 24 h. The final catalyst was denoted as RCOOH/stellate.

### Catalyst characterisation

Scanning electron microscopy (SEM) was conducted using a Zeiss Ultra 55 FEG-SEM microscope operating at 2 kV using InLens and Secondary electron imaging detectors and fitted with an energy dispersive X-ray spectrometer (EDX). Dispersed samples were drop cast from a methanol solution onto adhesive carbon discs on 12.5 mm aluminium stubs and splutter coated with a thin film (∼5 nm) of Pd : Au (80 : 20) to overcome charging. Transmission electron microscopy (TEM) micrographs were recorded at 200 kV on a FEI Tecnai G2 20 microscope fitted with a LaB_6_ source and a silicon drift EDX detector. Samples were dispersed in methanol and drop-cast onto a holey carbon support film on a copper grid. SEM and TEM images were analysed using ImageJ 1.53q software. Nitrogen adsorption/desorption isotherms were recorded at −196 °C on a Micromeritics ASAP 2060, after degassing at 200 °C for 10 h under vacuum. Surface areas were calculated using the Brunauer, Emmett, and Teller (BET) equation with the Barrett, Joyner, and Halenda (BJH) method applied to the desorption branch used to evaluate mesopore size distributions. X-ray photoelectron spectroscopy (XPS) was conducted using a Kratos Analytical Axis Ultra spectrometer fitted with a monochromated Al Kα (1486.7 eV) X-ray source, operating at 15 kV. CasaXPS Version 2.3.25PR1.0 was used for spectra calibration and fitting, with energy referencing to the C 1s peak of adventitious carbon at 284.8 eV. Acid site loadings were evaluated by NH_3_ pulse chemisorption on a Microtrac BELCAT II. Samples were pretreated at 150 °C under argon before pulse chemisorption of 10% ammonia in helium at 120 °C. For KCl titration, 50 mg of catalyst was mixed with 5 cm^3^ of aqueous KCl solution (0.1 M). The pH of the suspension was measured after 24 h using an Eutech Instruments PC 2700 pH meter until a stable pH value was obtained following the method by Molinero *et al.*^45^ The concentration of acid sites was then calculated using eqn (S1). Elemental analysis for sulphur quantification was carried out using a Thermo Scientific Flash 2000 Elemental Analyser. Thermogravimetric analysis (TGA) was carried out using a Shimadzu TGA-550 instrument. Samples were heated from 20 °C to 900 °C (ramp rate of 10 °C min^−1^) under flowing air (40 cm^3^ min^−1^). Solid-state NMR data was obtained using a Bruker NEO 700 MHz (16.4 T, ^1^H Larmor Frequency) NMR spectrometer with a 3.2 mm (^1^H/^31^P) MAS NMR probe, using a 3.2 mm ZrO_2_ rotor with Vespel rotor caps used under ambient conditions with 20 kHz MAS frequency. RF nutation frequencies of 83 kHz and 50 kHz were used for ^1^H and ^31^P, respectively. For {^1^H}–^31^P CP experiments, a 70–100% ramp was used for ^1^H to match 50 kHz ^31^P spin-locking. The ^1^H T_1_ was 0.5 seconds, but a recycle delay of 3 seconds was used to account for the probe duty cycle as 83 kHz SPINAL-64 ^46^^1^H heteronuclear decoupling was employed for 40 ms. 2048 transients were recorded and co-added. Chemical shifts were externally referenced to 85% liquid H_3_PO_4_ for ^31^P using ammonium dihydrogen phosphate (*δ*{^31^P} = 0.8 ppm). Spectra were analysed in Topspin 4.5.0.

### Catalysts screening

Fructose dehydration was performed in 30 cm^3^ reaction vials within an Anton Paar Monowave 400 microwave reactor. Catalyst (0.2 g) was added to an aqueous fructose solution (0.28 M, 10 cm^3^) and heated to the reaction temperature as quickly as possible (∼1 minute), and held for the desired reaction duration. Upon completion, flowing compressed air rapidly cooled (∼15 minutes) the external surface of the vial to 70 °C, before cooling to room temperature by quenching the vial in ambient temperature water for 3 minutes. The catalyst was recovered by centrifugation (4000 rpm, 5 minutes) using an Eppendorf 5702 centrifuge, and aliquots (0.2 cm^3^) of the liquid phase were diluted with deionised water (1 : 50 v/v) and filtered through a polyethersulfone syringe filter (0.2 µm) for high-performance liquid chromatography (HPLC) analysis. Quantitative HPLC analysis was conducted on an Agilent Infinity 1260 HPLC equipped with a refractive index detector (RID) and a diode array detector (DAD). Product resolution was achieved on a Bio-Rad Aminex HPX-87H ion exclusion column (300 mm × 7.8 mm), using a 0.01 N H_2_SO_4_ mobile phase (flow rate 0.6 cm^3^ min^−1^) under isothermal conditions (column temperature 50 °C).

Catalytic performance metrics are defined in the SI (eqn (S2) to (S5)). Sustainability metrics, including E-factor (with and without solvent) (eqn (S6) and (S7)), mass intensity (MI) (eqn (S8)), reaction mass efficiency (RME) (eqn (S9)), and energy economic coefficient (*ε*) (eqn (S10)), were evaluated to quantify material utilisation and to assess the overall efficiency of the reaction process. All green metrics are based on a single batch process of fructose dehydration to HMF in water. E-factor is defined as the mass of waste divided by the mass of product, with the mass of waste defined as either solely as the mass of humins, *i.e.* assuming the by-products (formic acid, levulinic acid, glucose, furfural, 1-(2-furyl)-2-hydroxyethanone) are valuable side products, or by defining HMF as the single valuable product and all by-products as waste. In addition, a comparison in which the solvent (water) is also considered as a waste stream is made based on the total mass of solvent used in the reaction solution, *i.e.* assuming that there are no losses *via* evaporation and that additional moles of water generated have a negligible impact. Mass intensity reflects the mass of fructose and catalyst used over the mass of HMF obtained, with the solvent (water) excluded due to its low environmental impact. Due to the facile recovery of the catalysts, the reaction mass efficiency is based solely on the mass of desired product (HMF) and the mass of substrate (fructose). Energy economy coefficient, *ε*, is calculated from the maximum HMF yield for each temperature, *i.e.* at differing time points, as opposed to a constant time point, to reflect optimal performance. These yields are normalised to the amount of energy used in each case.

For catalyst recycle tests, catalyst reactivation was achieved by repeating the activation step from the original synthesis, *e.g.* hydrochloric acid reflux for RPO_3_H_2_/stellate and hydrogen peroxide for RSO_3_H/stellate and RCOOH/stellate. For RPO_3_H_2_/stellate, the dried recovered spent catalyst was refluxed in concentrated hydrochloric acid (37%, 5 cm^3^) for 24 h, before isolation by centrifugation (8000 rpm, 0.5 h) and washing with deionised water to pH 7. The solid was dried overnight at room temperature prior to further drying at 80 °C for 24 h. The dried spent RSO_3_H/stellate catalyst was stirred in an aqueous hydrogen peroxide solution (9 cm^3^) for 24 h at room temperature, before isolation by centrifugation (8000 rpm, 0.5 h). The solid was washed with deionised water in triplicate and dried overnight at room temperature prior to further drying at 80 °C for 24 h. For RCOOH/stellate, the dried spent catalyst was stirred in an aqueous hydrogen peroxide solution (9 cm^3^) for 24 h at room temperature. The solid was isolated by centrifugation (8000 rpm, 0.5 h), washed with deionised water in triplicate and dried overnight at room temperature prior to further drying at 80 °C for 24 h.

## Results and discussion

### Catalyst characterisation

**Fig. 1:**
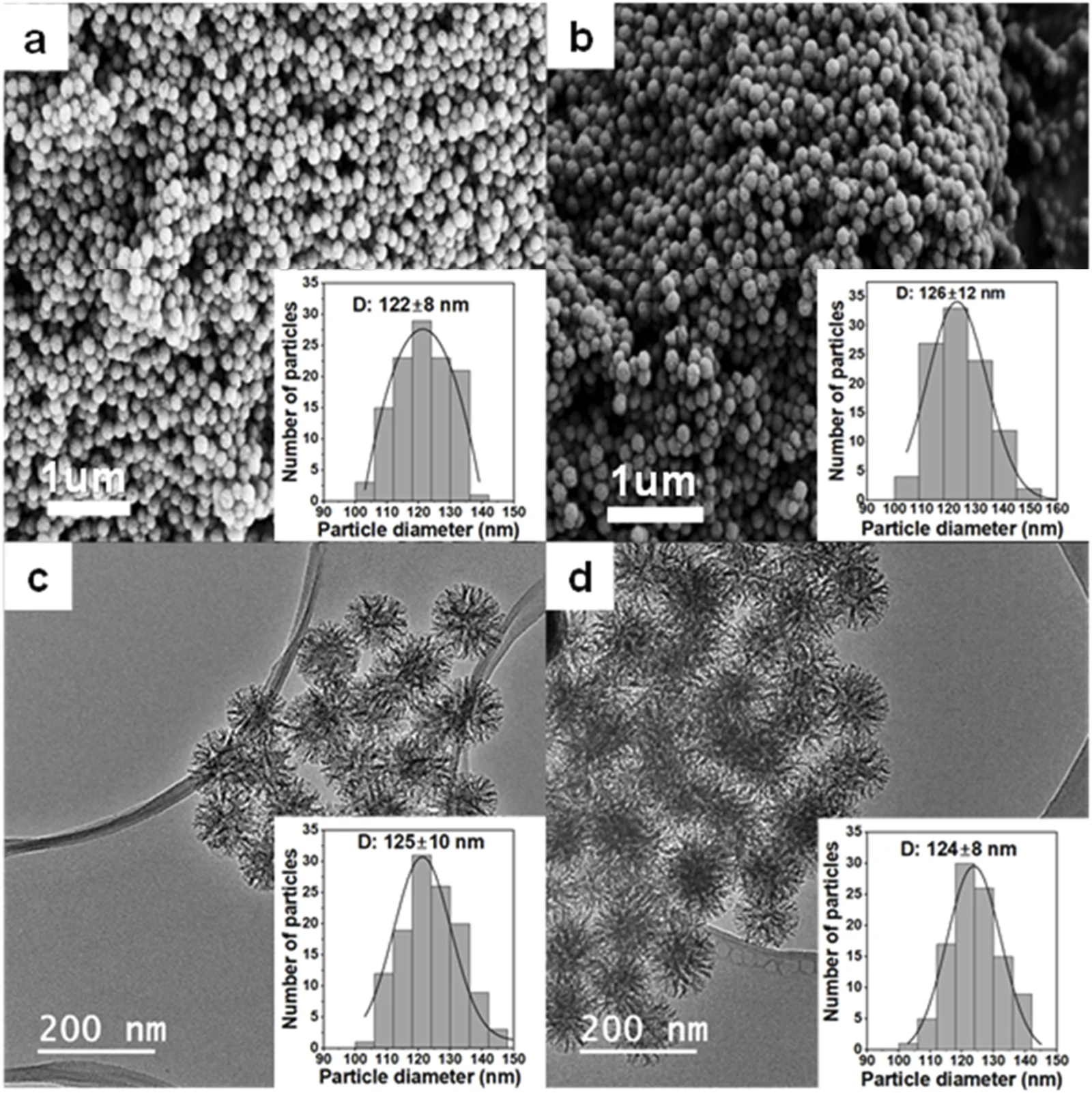
SEM (a) and (b) and TEM (c) and (d) images and corresponding particle size distributions of stellate silica (a) and (c), and RSO_3_H/stellate (b) and (d).

Electron micrographs, for both SEM and TEM, are shown in Fig. 1 and S1, confirming the successful formation of the desired stellate silica porous nanospheres, and the retention of this porous architecture post active site grafting. The distinctive star-like radial architecture and open pore morphology, originating from a central core, are clear by TEM, whilst spherical particle shape and size uniformity, and an absence of particle aggregation or secondary structures, are verified by SEM. The average particle sizes, reported in Table 1, are consistent with the literature,^44^ with high uniformity across all four materials apparent from deviation being less than 5%; thus, no impact from the introduction of acid sites arises.

**Table 1:** Textural properties of the silica nanosphere support and synthesised catalysts

Sample	BET surface area/m^2^ g^−1^	Ave. BJH pore diameter/nm	Ave. particle diameter SEM/nm	Ave. particle diameter TEM/nm
Stellate	468 ± 23	12.6 ± 2	122 ± 8	125 ± 10
RSO_3_H/stellate	503 ± 25	13.2 ± 2	126 ± 12	124 ± 8
RPO_3_H_2_/stellate	360 ± 18	13.4 ± 2	118 ± 7	122 ± 7
RCOOH/stellate	451 ± 23	11.8 ± 2	117 ± 7	121 ± 9

**Fig. 2:**
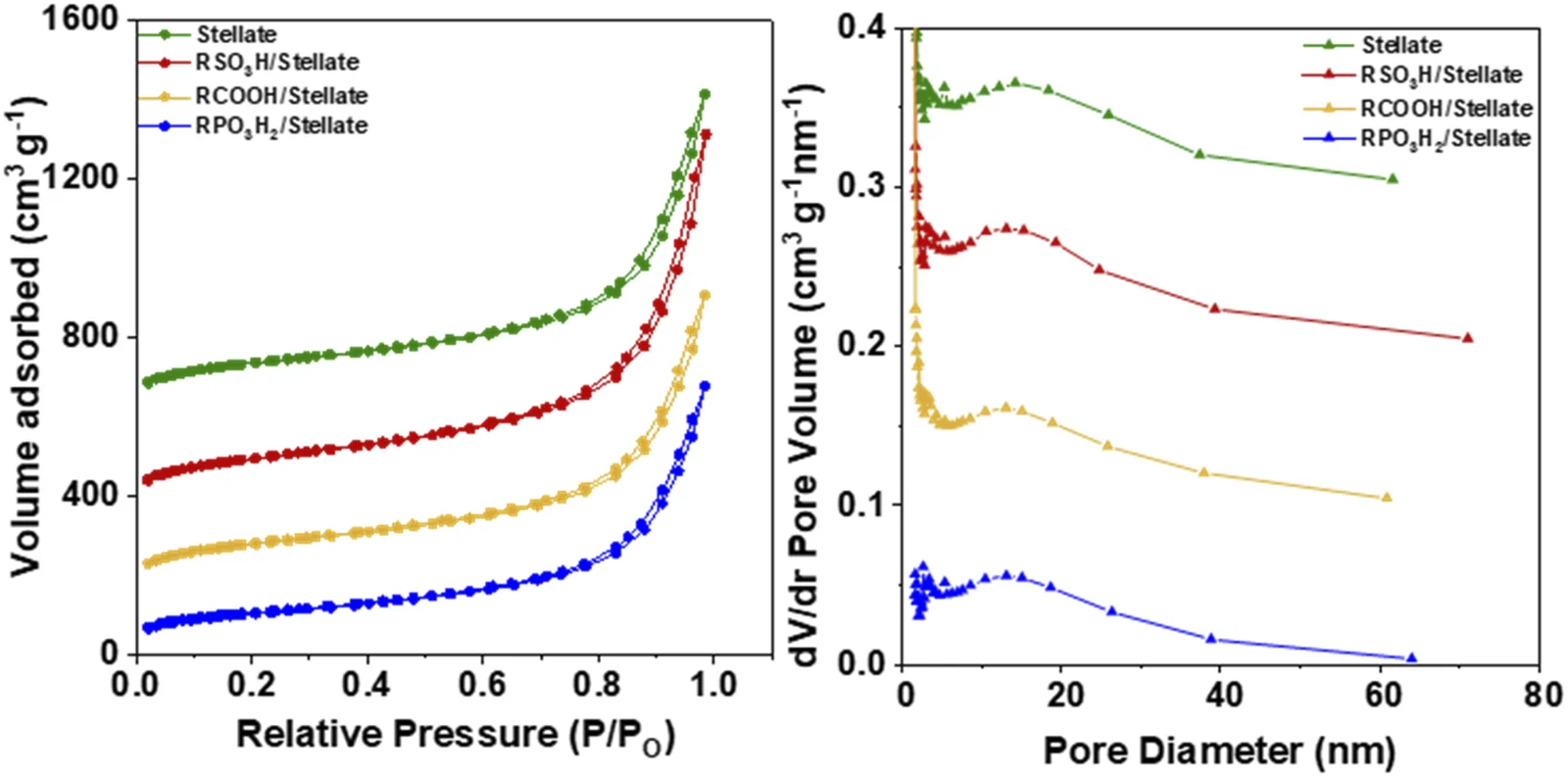
(a) Stacked nitrogen adsorption–desorption isotherms of stellate silica support and acid/stellate catalysts, data offset by 150, 350, and 600 cm^3^ g^−1^ for RCOOH/stellate, RSO_3_H/stellate, and stellate support, respectively, to aid visualisation. (b) Stacked BJH pore size distribution of stellate silica support and acid/stellate catalysts, data offset by 0.1, 0.2, and 0.3 cm^3^ g^−1^ nm^−1^ for RCOOH/stellate, RSO_3_H/stellate, and stellate support, respectively, to aid visualisation.

Stellate silica nanospheres exhibit a characteristic type IV nitrogen adsorption isotherm with capillary condensation at high relative pressures, as depicted in Fig. 2a, indicative of well-defined large mesoporosity and consistent with the TEM analysis. Acid grafting shows no significant impact on the nitrogen adsorption isotherms of the catalysts; however, close analysis *via* BET surface area comparisons reveals that for RPO_3_H_2_/stellate, a decrease is apparent, with a drop of 23% relative to the parent silica. This coincides with a higher acid loading (relative to the other acids) discussed below, *i.e.* high incorporation of grafted species results in a drop in surface area.^47^ In contrast, the surface areas of RSO_3_H/stellate and RCOOH/stellate are consistent with the parent support, and thus the lower loading of grafted species has no impact. Similar drops in surface area to those for RPO_3_H_2_/stellate have been reported when using water as the solvent for grafting sulphonic acid sites, with the change in solvent leading to an order of magnitude increase in active sites grafted.^15^ Thus, grafting in ethanol, as confirmed by TEM and nitrogen adsorption, preserves the structural integrity of the stellate architecture. The mesopores of the parent stellate silica skeleton (Fig. 2b) exhibit a broad size distribution, consistent with the literature and TEM, with the majority spanning ∼7–30 nm in diameter and an average size of 12.6 ± 2 nm (Table 1). Acid functionalisation impacts neither average size nor range of size distribution; however, the amount of nitrogen adsorbed, especially in the case of RPO_3_H_2_/stellate, does decrease, consistent with the (high) loading of active species within the pores.

The successful incorporation of organo-functionality (sulphonic, phosphonic, and carboxylic groups) is confirmed by XPS analysis (Fig. S2), with binding energies consistent with the literature values for sulphonic,^15^ phosphonic(phosphonates),^48^ and carboxylic acids.^49^ The sulphonic and phosphonic systems present as a single doublet. For the former, this confirms the complete conversion of the grafted precursor to the desired acid following the hydrogen peroxide treatment, *i.e.* a significant shift relative to the thiol binding energy.^15^ However, for the latter, decoupling the phosphorus signal from phosphorus present in the precursor (R–P(O)(OC_2_H_5_)_2_) *vs.* phosphorus present in the acid derivative (R–P(O)(OH)_2_) is unfeasible by XPS due to a highly similar environment, and thus comparable binding energies of the ejected photoelectrons. Solid-state ^31^P NMR spectroscopy of RPO_3_H_2_/stellate addresses this limitation (Fig. S3), revealing two distinct phosphorus environments, with chemical shifts of 23 and 32 ppm. The dominant signal at 32 ppm is assigned to the phosphonic group bound as C–P(=O)(OH)_2_ in a silane-type environment on silica, while the smaller second peak at 23 ppm is consistent with monodentate, phosphate-like silica supported species, *i.e.* unactivated precursor.^50^ From peak integration, the phosphonic acid species account for ∼73% of the phosphorus present in the catalyst. In the case of carboxylic acid catalysts, the C 1s region is deconvoluted into three species, with a peak at 288.8 eV corresponding to the COOH functionality. XPS also facilitates quantitative analysis with loadings reported in Table 2, either from the total S and P content or from quantification of the COOH species from deconvolution of the C region. This assumes uniform deposition of organo-functionality throughout the silica skeleton, although given large mesopore diameters relative to the grafted species, this is reasonable. These loadings highlight the higher efficiency of diethylphosphatotriethoxysilane grafting over the other alkoxysilanes, which justifies the low surface area and N_2_ adsorption discussed earlier. Whereas atomic loadings for S and C, present as carboxylic acid, are consistent with each other, which is likewise reflected in the N_2_ adsorption analysis. A 0.4 wt% sulphur loading from CHNS bulk elemental analysis for RSO_3_H/stellate is in good agreement; thus, comparable loading from surface and bulk analysis is indicative that grafting is uniform throughout the support.

**Table 2:** Acid site loading of the synthesised catalysts

Sample	XPS elemental loading/atom% (wt%)	Acid^a^/mmol g^−1^	Acid^b^/mmol g^−1^
RSO_3_H/stellate	0.63 (0.97)	0.041	0.079
RPO_3_H_2_/stellate	2.06 (3.09)	0.097	0.151
RCOOH/stellate	0.59 (0.30)	0.021	0.014

a From NH_3_ pulse titrations.

b From KCl titrations.

Acid site loadings are quantified directly by NH_3_ and KCl titrations (Table 2), in part to mitigate the issues arising from the phosphorus XPS analysis. The higher loadings for RPO_3_H_2_/stellate are attributed to a combination of the higher grafting efficiency in RPO_3_H_2_/stellate and its diprotic nature. Acid site quantification enables acid site performance to be evaluated, as any evaluation based on XPS would be redundant due to the uncertainty of the contribution to the phosphorus signal from the presence of residual (unactivated) organo-acid precursor (diethylphosphatotriethoxysilane). The two other catalysts (RSO_3_H/stellate and RCOOH/stellate), in the case of the more common approach of NH_3_ titrations, show comparable loadings. While the acid site loadings of RSO_3_H/stellate are lower than previous reports by Pirez *et al.*,^51^ and Price *et al.*,^15^ these reported higher XPS sulphur loadings, with similar sulphur to acid site ratios across all three studies. We point out that the higher loadings previously reported reflect the use of a higher surface area support, namely SBA-15,^47,51^ or an aqueous grafting protocol.^15,47^

With respect to the acid strengths of the functional groups, convention would be to conduct NH_3_ temperature programmed desorption (TPD). However, the presence of organic functionality renders thermal analytic techniques redundant, as the active species decompose at comparable temperatures to NH_3_ desorption. However, the acid strength can be referenced based on their organic acid analogues, with the trend of acid strength being RSO_3_H/stellate > RPO_3_H_2_/stellate > RCOOH/stellate. This being based on p*K*_a_ values of 1.92 (propane-1-sulfonic acid), 2.49 and 8.18 (propane-1-phosphonic acid), and 4.87 (propanoic acid) as approximate values for RSO_3_H/stellate, RPO_3_H_2_/stellate, and RCOOH/stellate, respectively.

### Catalytic activity

**Fig. 3:**
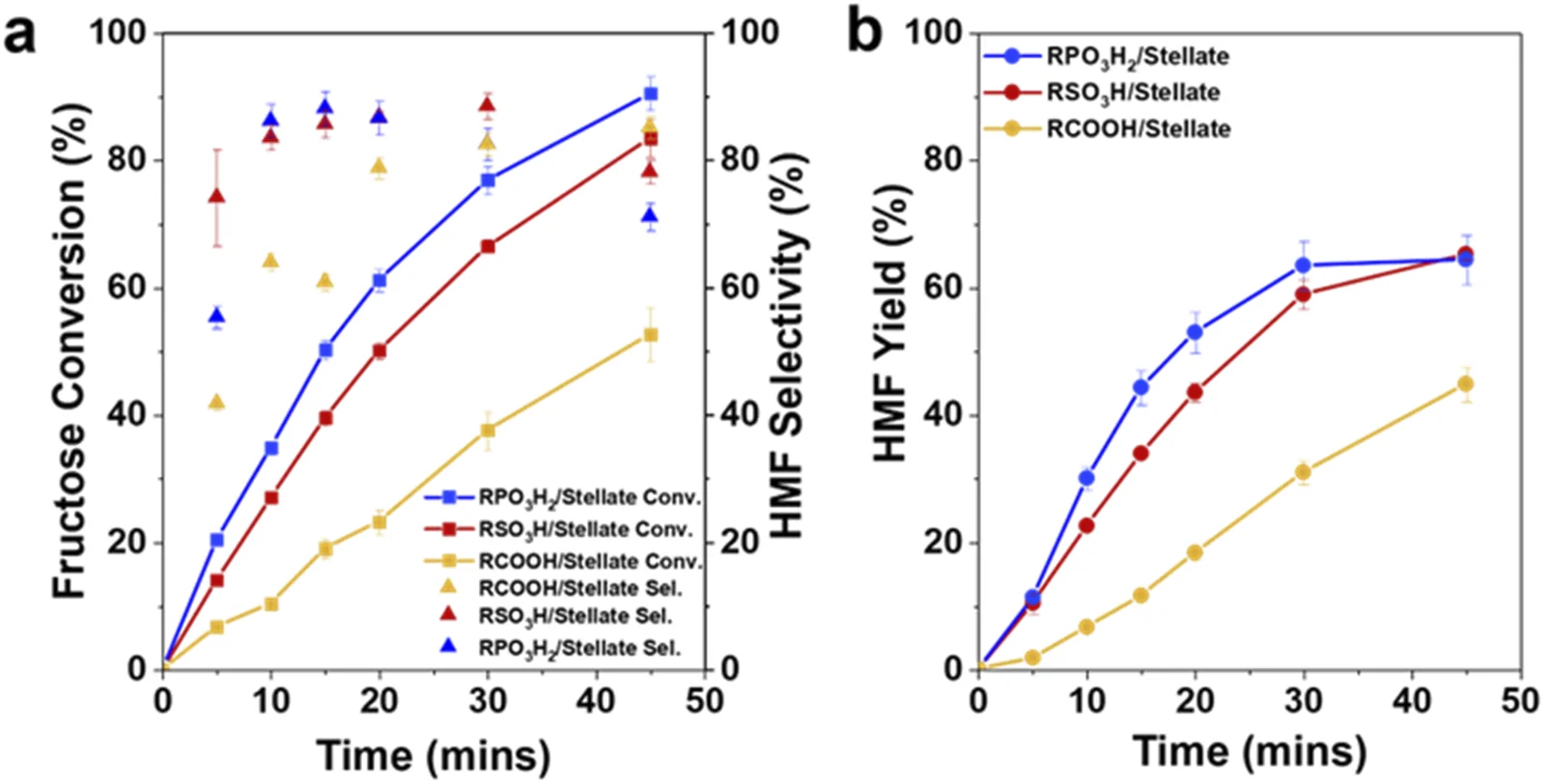
Catalytic performance data for fructose dehydration to HMF at 165 °C showing (a) fructose conversion and HMF selectivity reaction profiles, and (b) HMF yield profiles. Reaction conditions: 10 cm^3^, 0.28 M aqueous fructose solution, 0.2 g catalyst, 165 °C, 900 rpm, microwave irradiation.

Catalyst performance in monophasic aqueous phase fructose dehydration at 165 °C is illustrated in Fig. 3. Increased acid strength in both RPO_3_H_2_/stellate and RSO_3_H/stellate leads to higher initial activity and optimal HMF selectivity, with rates for HMF formation of 5.1 and 4.0 mmol h^−1^ over the initial 0.17 h, respectively. These reflect a ∼4.3-fold and ∼3.3-fold higher rate relative to RCOOH/stellate (1.2 mmol h^−1^). However, acid strength is not solely the deciding factor in activity, as despite not being the strongest acid (based on the p*K*_a_ value discussed earlier), RPO_3_H_2_/stellate outperform the more acidic RSO_3_H/stellate, due to the higher number of acid sites. Given the diminished initial performance when the p*K*_a_ of the acid increases to 4.87 (carboxylic acid), the impact of the diprotic nature of RPO_3_H_2_/stellate is negligible, with activity dictated by the more acidic proton. While the stronger acids do initially show high selectivity, around ∼85–90% at up to 20 min, extended reaction times (45 min) see these drop, consistent with the established concepts that HMF selectivity is inversely proportional to acid strength, *i.e.* strong acidity results in higher reaction rates but also facilitates catalysis of side reactions and formation of polyfuranics (humins).^15,19^ This trade-off is mirrored if the number of acid sites within the reaction is elevated, though an increase in the mass of catalysts used.^52^ In contrast, selectivity at extended reaction times for the weaker RCOOH/stellate catalyst remains high, suggesting less humin formation, further confirmed by a reduced yellow/brown colour present in the final reaction solution.

As expected, increasing process temperature to 175 and 185 °C, as shown in Fig. S4, results in faster fructose conversion kinetics, albeit at the expense of HMF selectivity. The rate and extent of the impact, both positive (conversion) and negative (selectivity), are strongly dependent on acid strength.^53^ The influence of the strongest acids, RPO_3_H_2_/stellate and RSO_3_H/stellate, is more extreme than that of the weaker RCOOH/stellate catalyst, which, in general, shows the highest HMF selectivity at these elevated temperatures.

In homogeneous catalytic systems, Körner *et al.*^18^ showed that aqueous phosphoric, sulphonic and acetic acids can achieve comparable yields, ∼45%, ∼40%, and ∼45%, respectively, through governance over pH and reaction length. So for both homogeneous and heterogeneous systems, acid strength alone does not dictate HMF yields, with reaction parameters also critical in balancing between HMF and by-product formation, *via* side reactions. Under microwave heating in aqueous media, Kong *et al.*^42^ reported ∼88.3% HMF yield at 186 °C in 10 min over sulphonated carbon microsphere catalyst, albeit at a higher acid site to fructose ratio, whilst here RSO_3_H/stellate reaches ∼95% fructose conversion (∼72% HMF selectivity) under comparable reaction temperature. However, the study by Kong *et al.* resulted in a slightly lower energy efficiency (Table S1). The significant benefit of microwave heating is further apparent when benchmarking against a similar RSO_3_H/stellate catalyst under conventional heating, which after 6 h only reaches ∼58% conversion, albeit at 120 °C.^15^

**Fig. 4:**
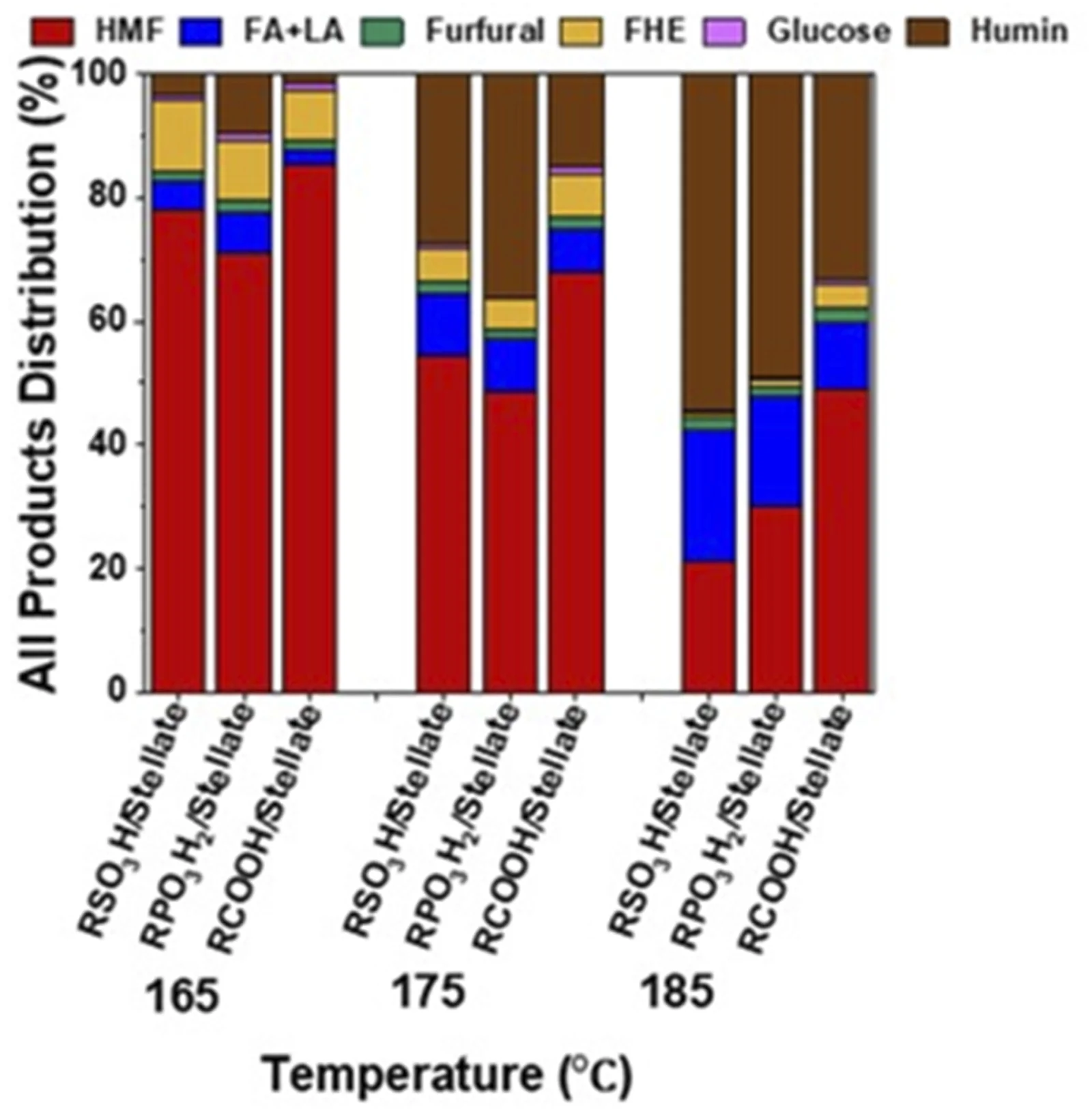
Complete product distribution from aqueous fructose dehydration to HMF at 0.75 h. Reaction conditions: 10 cm^3^, 0.28 M aqueous fructose solution, 0.2 g catalyst loading, temperatures of 165 °C, 175 °C, and 185 °C, 900 rpm, microwave irradiation.

Detailed evaluation of all products formed as a function of process temperature and catalytic system employed are shown in Fig. 4, with by-product yield profiles detailed in Fig. S5–S7. Major non-humin by-products are comprised of formic and levulinic acid (FA and LA, respectively), which arise from acid-catalysed HMF rehydration at elevated temperatures,^20^ and 1-(2-furyl)-2-hydroxyethanone (FHE), also known as hydroxyacetylfurane. While the latter is less deliberate within fructose dehydration studies, it can result from the rehydration and rearrangement of a 2,3-enediol intermediate produced *via* acyclic 2,3 enolisation of fructose.^54^ Minor by-products, glucose and furfural, result from either acid-catalysed isomerisation or acid-catalysed carbon–carbon cleavage and subsequent dehydration, respectively.^55^

Humins represent a significant additional undesirable by-product, for which higher temperatures accelerate the C–C cleavage and aldol condensation mechanisms,^56^ exacerbating the situation. In conjunction, the nature of the acid catalyst employed (for fructose dehydration) also strongly influences the evolution and solubility of humins formed. Thus, increasing either or both acid strength and reaction temperature results in favourable conditions for the generation of humin by-products.^57,58^ Humins result from the aldol condensation of HMF, reportedly *via* a hydration intermediate, 2,5-dioxo-6-hydroxyhexanal (DHH),^59^*via* self (reaction with HMF) and cross (reaction with fructose) condensation, with DHH resulting from acid-catalysed rehydration of HMF.^60^ Stronger acids and elevated temperatures accelerate this reaction pathway,^13,15^ whilst milder acids such as carboxylic acids are less likely to promote these condensation and rehydration reactions, resulting in slower humins formation.^18^ At 185 °C, the lowest proportion of humins (∼33%) was obtained when RCOOH/stellate, whereas RPO_3_H_2_/stellate and RSO_3_H/stellate generated increasingly more, consistent with stronger acids enhancing formation.^61^ At the two lower temperatures, RPO_3_H_2_/stellate produces the greater levels of humins, which is accounted for by its higher acid site loading. Thus, at lower temperatures, acid site loading dictates, whereas at high temperatures, acid strength governs. Close inspection of HMF yields (Fig. 3b) reveals a plateauing or even diminishing levels with increasing reaction time, with the impact following the trend of RSO_3_H/stellate ≈ RPO_3_H_2_/stellate > RCOOH/stellate. This is indicative of self-condensation being favourable to cross-condensation.^15^ A second key observation from the study at 185 °C is that all catalysts achieve comparable optimal HMF yields, with acid strength primarily affecting the time needed to reach these yields.^18^

**Fig. 5:**
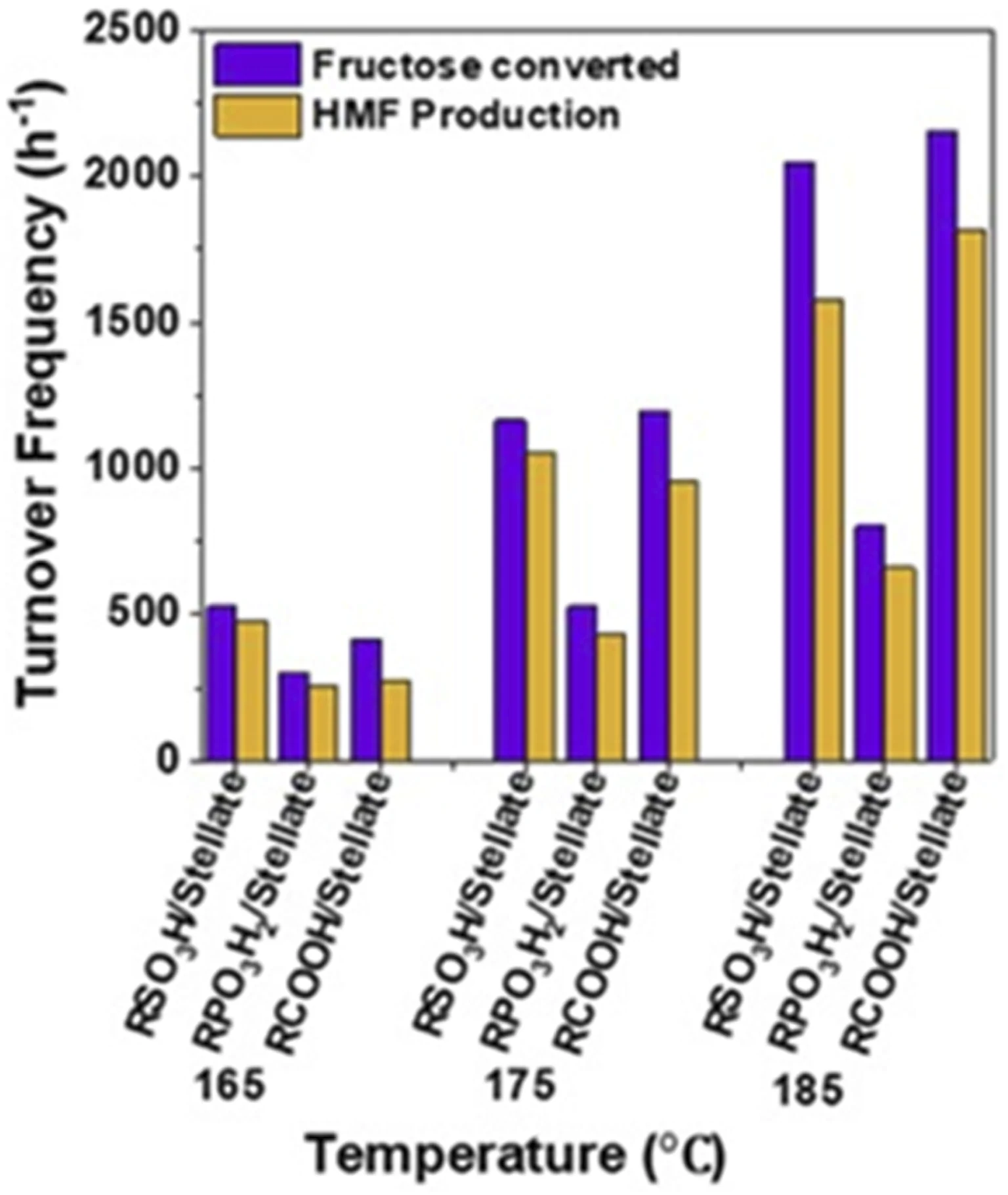
TOFs of fructose consumption and HMF production, based on initial rates calculated over the first 0.17 h and acid site loadings from NH_3_ titrations.

To provide a more comprehensive assessment of catalyst performance, further analysis of the reaction kinetics, focusing on the inherent activity of the different active species, was provided through turnover frequencies (TOFs) of fructose consumption and HMF formation (Fig. 5). Irrespective of reaction temperature, the phosphonic acid sites exhibit significantly lower performance per active site, whilst the performance of the sulphonic and carboxylic systems is relatively comparable. This apparent lower site activity of RPO_3_H_2_/stellate likely reflects its diprotic nature. As outlined above, NH_3_-TPD is not feasible, so deconvolution of strong and weak sites is not an option; however, adjusted TOFs, based on assuming only 50% of the acid site loadings from NH_3_ result from the stronger acid sites, would put this system closer to the other catalytic material.

Performance is temperature sensitive; at the lower temperature, RSO_3_H/stellate is preferable, which is put down to its stronger acid strength, whereas at the higher temperature, RCOOH/stellate outperforms, due to reduced side reactions and humin formation (and poisoning). HMF production TOFs of 16 h^−1^ have been reported from a comparable sulphonic acid functionalised on stellate under conventional heating at 120 °C, whilst the same sites on a hierarchical mesoporous micropores zeolite support at 160 °C in a biphasic reaction system resulted in a TOF of 2760 h^−1^, with the contribution from sulphonic sites being 1686 h^−1^.^33^ The employment of an optimal biphasic reaction medium at least partially accounts for the higher TOFs *vs.* ∼482 h^−1^ for RSO_3_H/stellate. In comparison, a TOF of 259 h^−1^ is witnessed for RPO_3_H_2_/stellate, which is favourable when benchmarked against 11.7 h^−1^ for zirconium phosphate at 240 °C.

**Fig. 6:**
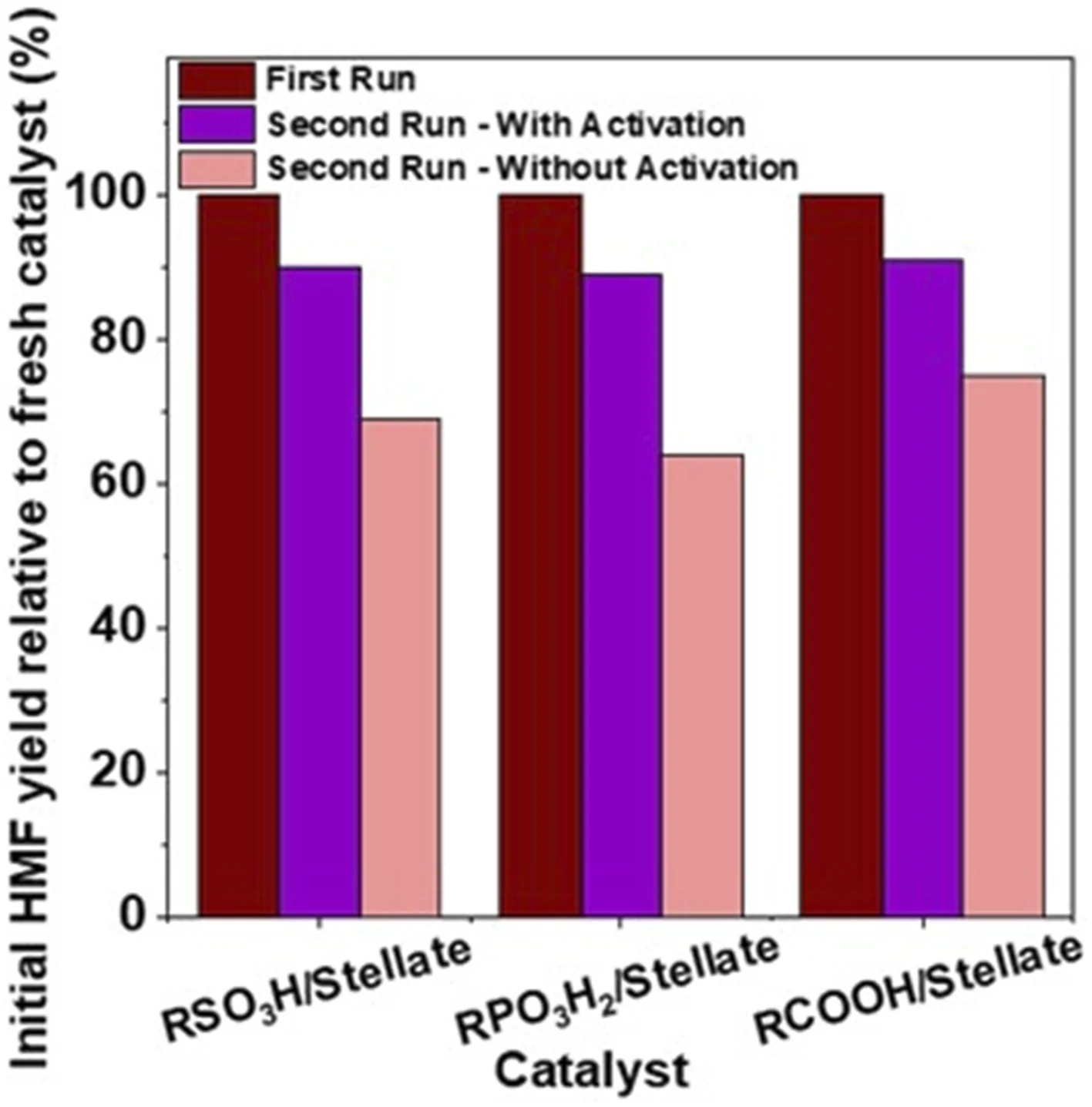
Initial HMF yield relative to fresh catalyst of RSO_3_H/stellate, RPO_3_H_2_/stellate, and RCOOH/stellate catalysts over two cycles, with and without catalyst regeneration. Initial reaction conditions: 10 cm^3^, 0.28 M fructose solution, 2 wt% catalyst, at 165 °C, 900 rpm for 45 min.

Humin formation during the reaction is further apparent from the appearance of the recovered catalysts after a reaction (Fig. S8) and *via* thermogravimetric analysis (TGA) in Fig. S9–S13. All recovered catalysts exhibited significant browning, indicating deposition of humin by-products, which are confirmed by TGA. A substantial mass loss was observed at ∼500 °C, corresponding to thermal oxidative degradation of the highly cross-linked structure of the deposited humins.^62^ Regardless of reaction temperature, RPO_3_H_2_/stellate exhibited the largest mass loss, consistent with a strong acid nature and high acid site loading.^52,63,64^ In comparison, the weaker RCOOH/stellate (especially true at 185 °C) showed substantially lower humins.^65^ The impact of humins deposition (fouling) and requirements to mitigate these are apparent from recycle studies conducted at 165 °C. To counteract the undesirable catalyst fouling, reactivation, *via* subjecting the spent catalysts to the initial activation process, was shown to be highly efficient, returning performance to 90% of the fresh catalyst (Fig. 6), although the process does not return the catalyst to its original freshly prepared white state (Fig. S14), suggesting only partial removal of surface deposits. Thus, a reactivation protocol is critical prior to reuse,^66^ akin to requirements reported for other acid catalysts, where humin deposits transpire.^67,68^ Consequently, catalyst deactivation, *via* surface fouling, and challenging catalyst performance recovery are key limitations of the monophasic aqueous system,^69^ for which biphasic reaction media can mitigate by improving product separation and catalyst recovery.^21^

While the sustainability of a process is commonly implied when feedstocks are biomass-derived, and processes employ alternative heating/energy sources or catalysis, the application of green metrics to assess the systems quantitatively is paramount.^70^ To assess the three catalytic systems and the role of process temperature and time calculations, metrics focusing on waste produced, mass, and energy balances are reported in Table S1. The sulphonic acid system is generally shown to be preferable at lower reaction temperatures, reflecting optimal yields whilst not being compromised by excessive humin formation. In contrast, at the higher temperature, the stronger acidity of RSO_3_H/stellate is detrimental, with the weaker acid systems, in the case of mass and waste based metrics, optimal when compared at maximum HMF yields for each catalyst, due to great process selectivity. This is more apparent at 185 °C, when comparing catalyst performance at a constant time point of 0.75 h, for which greater humin and waste by-product formation significantly impact the acidic systems (RSO_3_H/stellate and RPO_3_H_2_/stellate). However, with respect to the energy economic coefficients, which assess power requirements, RSO_3_H/stellate is shown to be favourable irrespective of temperature due to obtaining optimal HMF yields the quickest. Thus, aqueous phase HMF dehydration process performance requires a balancing of catalyst strength and reaction conditions (time and temperature), if maximum HMF yields are to be achieved.

## Conclusion

Advancement of catalytic materials for lignocellulosic biomass conversion to HMF is key to enhancing process activity and selectivity. In this work, stellate silica, with its distinctive porosity and expansive surface area, has been successfully implemented as a support architecture for the organo-functionalisation of three diverse acidic groups, namely sulphonic, phosphonic and carboxylic. Introducing these acid functionalities provides a spectrum of acid site strengths and properties, with the nature of acid functionality significantly influencing the catalytic performance towards fructose dehydration to HMF, for which reaction time and temperature also play a role. Catalyst performance was systematically investigated in an aqueous system and under microwave irradiation. While the strongest acid (SO_3_H/stellate) led to a ∼62% yield within 5 min at 185 °C, and a TOF of 1578 h^−1^, reflecting optimal initial activity, extended reaction times revealed a distinct advantage for RCOOH/stellate, which achieved superior HMF selectivity (∼49%) at 185 °C compared to their RSO_3_H/stellate (∼21%) and RPO_3_H_2_/stellate (∼30%) counterparts. Despite its weaker acidity, RCOOH/stellate exhibits the highest TOF for HMF formation, reaching up to 1818 h^−1^, revealing that strength is not everything, with weaker acidity retaining high activity due to reduced deactivation. In contrast, at 165 °C, RSO_3_H/stellate is optimal for the manufacturing of HMF; thus, at this lower temperature, deactivation of the strong site is less of an issue, at least for the initial 10 min of the reaction. Increased reaction times result in deactivation in all systems; however, through relatively facile reactivation processes, room temperature H_2_O_2_ treatment (RSO_3_H/stellate and RCOOH/stellate) or the harsher HCl under reflux (RPO_3_H_2_/stellate), restoration of 90% of the original catalytic performance is achieved. Another key benefit of these catalytic materials.

## Author contributions

Aina Syahida Jamal: investigation, methodology, formal analysis, data curation, writing – original draft, writing – review & editing. Cameron-Alexander H. Price: conceptualisation, investigation, methodology. Molly Smith: investigation, methodology. Daniel Lee: supervision, investigation. Jesús Esteban: investigation, writing – review & editing, supervision, funding acquisition. Christopher M. A. Parlett: conceptualisation, investigation, methodology, writing – review & editing, supervision, funding acquisition.

## Conflicts of interest

The authors declare that they have no known competing financial interests or personal relationships that could have appeared to influence the work reported in this paper.

## Supplementary Material

SE-010-D6SE00627B-s001

## Data Availability

The raw data that support the findings of this study are openly available in Figshare at https://doi.org/10.48420/c.8645955. All data that support the findings of this study are included within the article (and any supplementary information (SI) files). Supplementary information is available. See DOI: https://doi.org/10.1039/d6se00627b.
