# Supplementary material for: Fructose dehydration to 5-hydroxymethylfurfural over supported acid catalysts: impact of acid chemical species
Source: Sustain Energy Fuels. 2026 Aug 5;10(18):4347–59. doi: 10.1039/d6se00627b (PMC13576675; doi:10.1039/d6se00627b)
Supplement: Supplementary file 1 [file SE-010-D6SE00627B-s001.pdf]

Supplementary Information (SI) for Sustainable Energy & Fuels.
This journal is © The Royal Society of Chemistry 2026

ESI: Fructose dehydration to 5-hydroxymethylfurfural over supported acid catalysts: Impact of acid chemical species

Aina Syahida Jamal[1], Cameron-Alexander H. Price[1,2], Molly Smith[1], Daniel Lee[1], Jesús Esteban[1,2], Christopher M.A. Parlett[1,2,4,]*

1. *Department of Chemical Engineering, The University of Manchester, Oxford Road, Manchester M13 9PL, United Kingdom.*
2. *UK Catalysis Hub, Research Complex at Harwell, Rutherford Appleton Laboratory, Harwell, Oxfordshire OX11 0FA, United Kingdom.*
3. *Department of Chemical Engineering and Materials, Faculty of Chemical Sciences, Complutense University of Madrid, 28040 Madrid, Spain.*
4. *University of Manchester at Harwell, Diamond Light Source, Harwell Science and Innovation Campus, Didcot, Oxfordshire OX11 0DE, United Kingdom.*

*christopher.parlett@manchester.ac.uk

Catalyst Characterisation

$$\frac{1000 \times 10^{-pH} \times 0.005\ [L]}{0.05\ (g)} = \frac{mmol\ Br\emptyset nsted\ groups}{catalyst\ mass\ [g]}$$

(Equation S1)

Determination of catalytic performance

For catalytic fructose dehydration, the conversion of fructose (%), the product yield (%), and product selectivity (%) are defined by Equations (S2) to (S4).

$$\text{Fructose conversion (\%)} = \frac{n_{fru,0} - n_{fru,t}}{n_{fru,0}} \times 100\ \% \qquad \textbf{(Equation S2)}$$

$$\text{Product yield (\%)} = \frac{n_{i,f}}{n_{fru,0}} \times 100\ \% \qquad \textbf{(Equation S3)}$$

$$\text{HMF selectivity (\%)} = \frac{n_{HMF}}{n_{fru,0} - n_{fru,t}} \times 100\ \% \qquad \textbf{(Equation S4)}$$

i denotes the final products, i.e., fructose, glucose, HMF, levulinic acid, formic acid, furfural, FHE; Fru denotes fructose; subscript t is the reaction time; subscript 0 denotes initial time (i.e., t = 0).

Turnover Frequency (TOF)

$$\text{TOF} = \frac{Rate\ of\ Fructose\ converted}{Number\ of\ active\ sites}\ \text{or}\ \frac{Rate\ of\ HMF\ produced}{Number\ of\ active\ sites}$$

(Equation S5)

Green Metric Calculation

E-factor (solvent recycled) = $\frac{Total\ mass\ of\ waste\ (excluding\ solvent)}{Mass\ of\ final\ product}$ **(Equation S6)**

E-factor (solvent not recycled) = $\frac{Total\ mass\ of\ waste\ (including\ solvent)}{Mass\ of\ final\ product}$ **(Equation S7)**

MI = $\frac{Total\ mass\ in\ process}{Mass\ of\ product}$ **(Equation S8)**

RME (%) = $\frac{Mass\ of\ product}{Total\ mass\ of\ reactants} \times 100$ **(Equation S9)**

ε = $\frac{HMF\ Yield\ (mass\ fraction)}{Energy\ (kJ)}$ **(Equation S10)**

Catalyst Characterisation

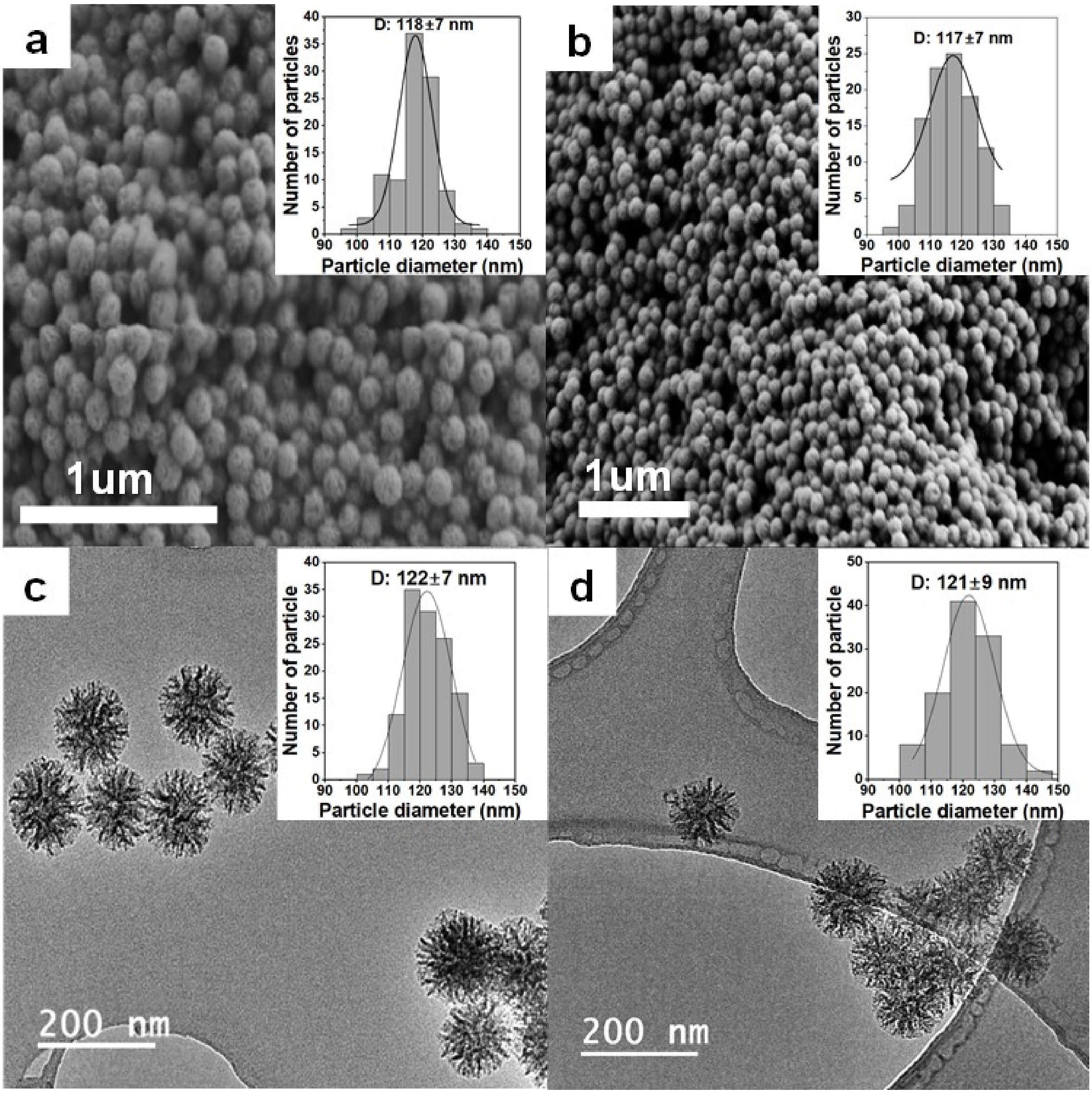

Figure S1. SEM (a) and (b) and TEM (c) and (d) images and corresponding particle size distributions of RPO_3H_2/Stellate (a) and (c), and RCOOH/Stellate (b) and (d).

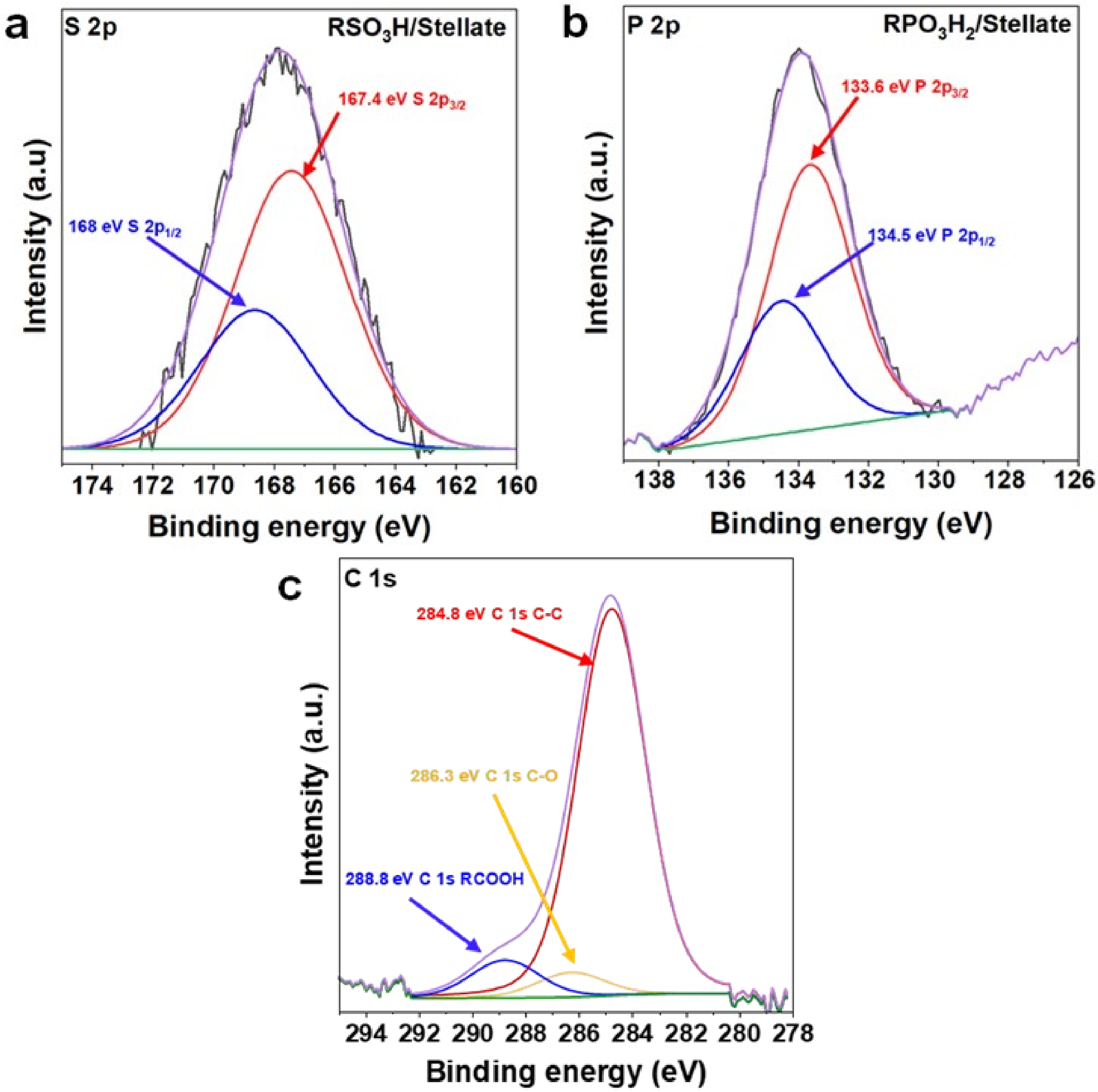

Figure S2. X-ray photoelectron spectra of (a) RSO_3H/Stellate, (b) RPO_3H_2/Stellate, and (c) RCOOH/Stellate.

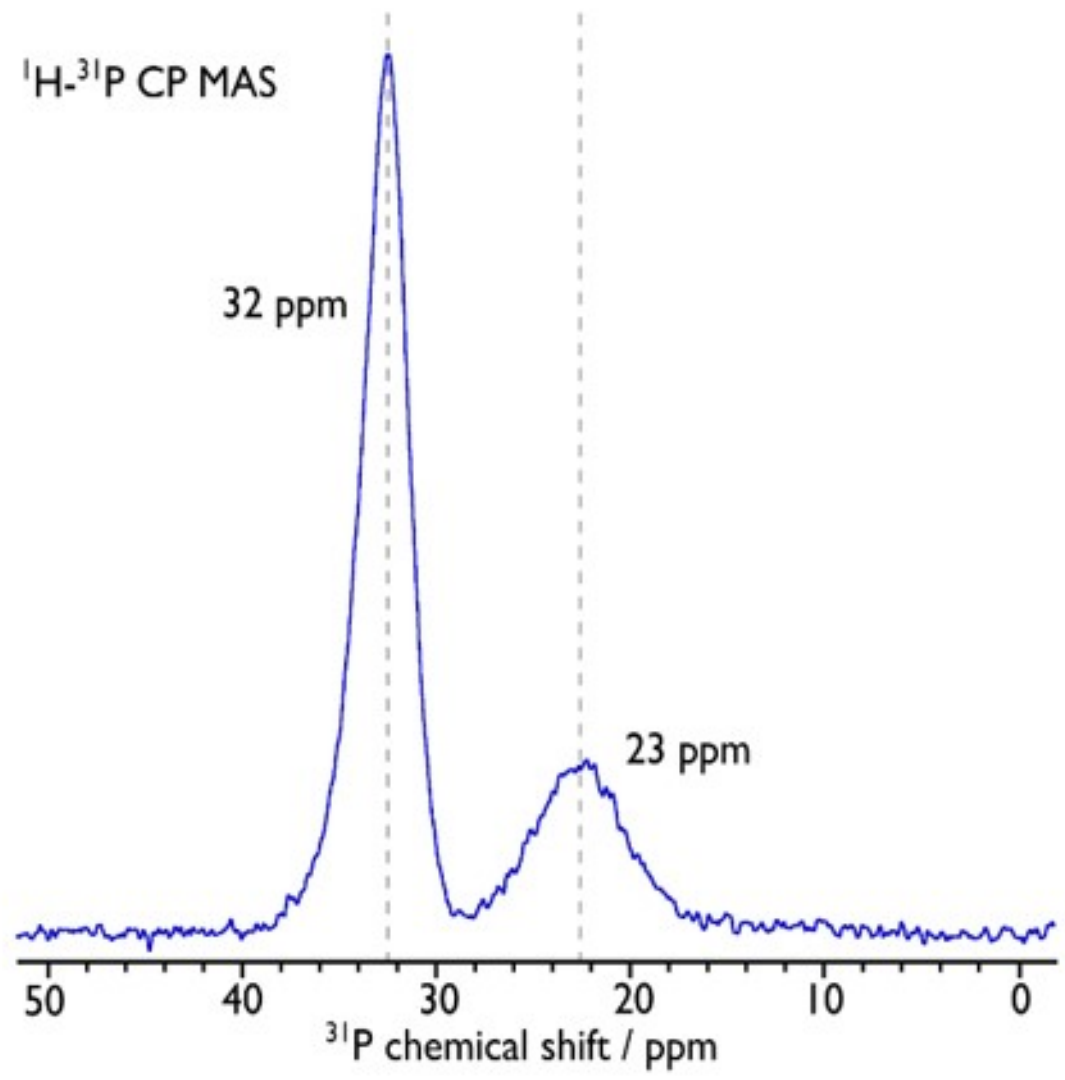

Figure S3. Solid-state {^{1}H-}^{31}P CPMASNMR spectrum of RPO_3H_2/Stellate.

Catalysts screening

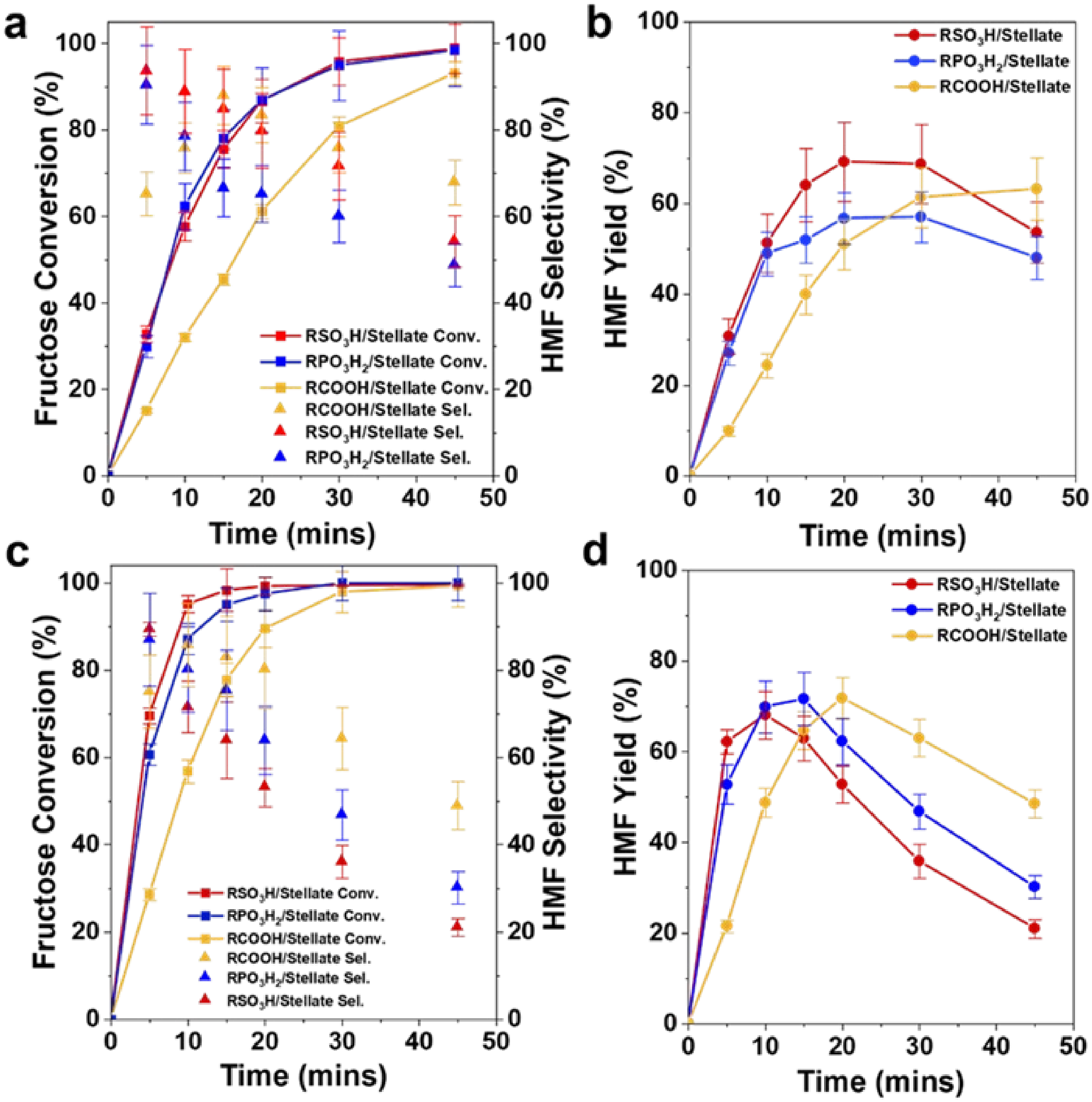

Figure S4. Catalytic performance data for fructose dehydration at 175 °C (a) and (b) and 185 °C (c) and (d), showing fructose conversion and HMF selectivity profiles (a) and (c), and HMF yield profiles (b) and (d). Reaction conditions: 10 cm^3, 0.28 M aqueous fructose solution, 0.2 g catalyst, 900 rpm, microwave irradiation. Conv. represents the fructose conversion, and Sel. represents the HMF selectivity.

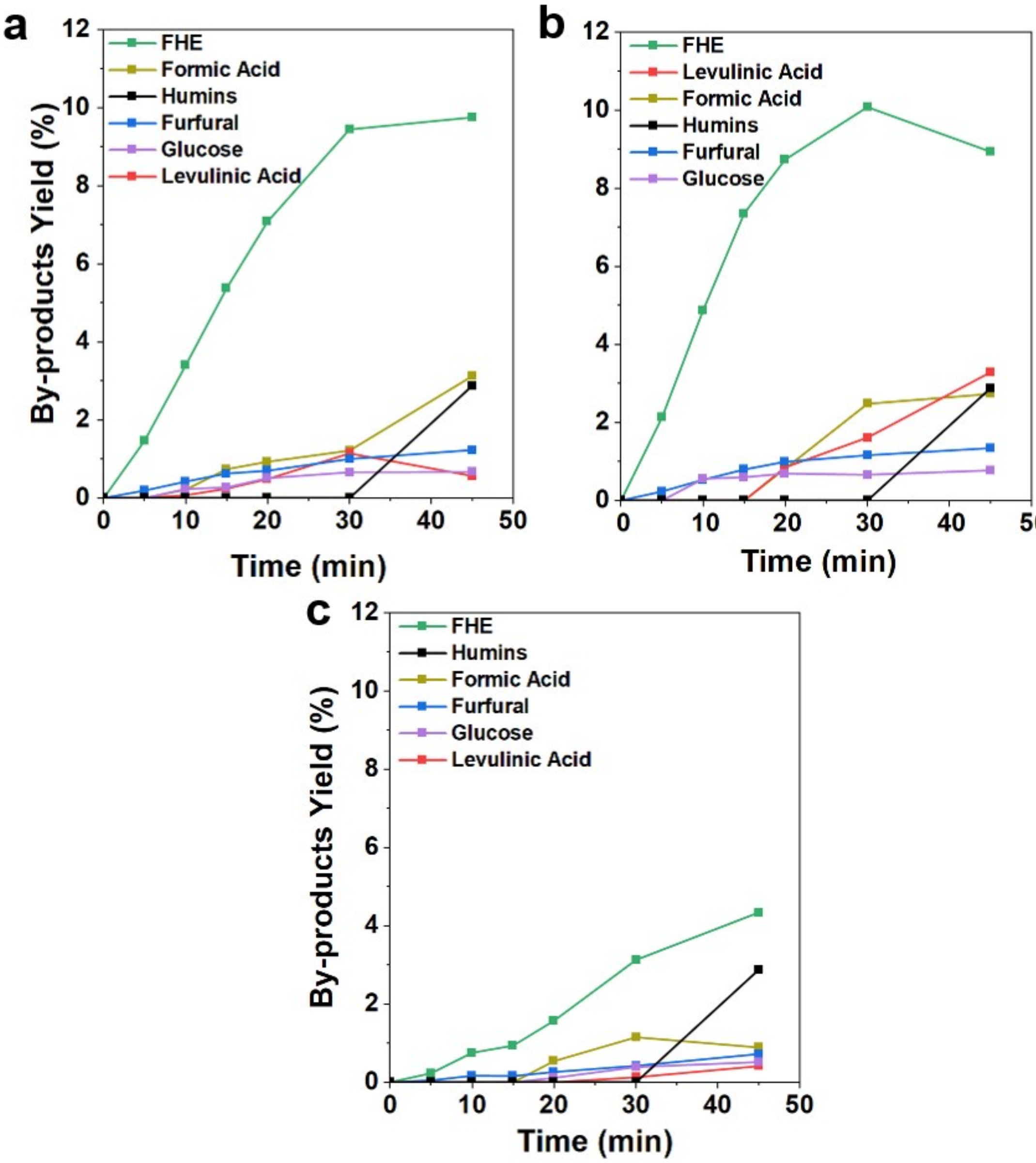

Figure S5. Comparison of by-product yield profiles for aqueous fructose dehydration at 165 °C over (a) RSO_3H/Stellate, (b) RPO_3H_2/Stellate, and (c) RCOOH/Stellate. Reaction conditions: 10 cm^3, 0.28 M aqueous fructose solution, 0.2 g catalyst, 900 rpm, microwave irradiation.

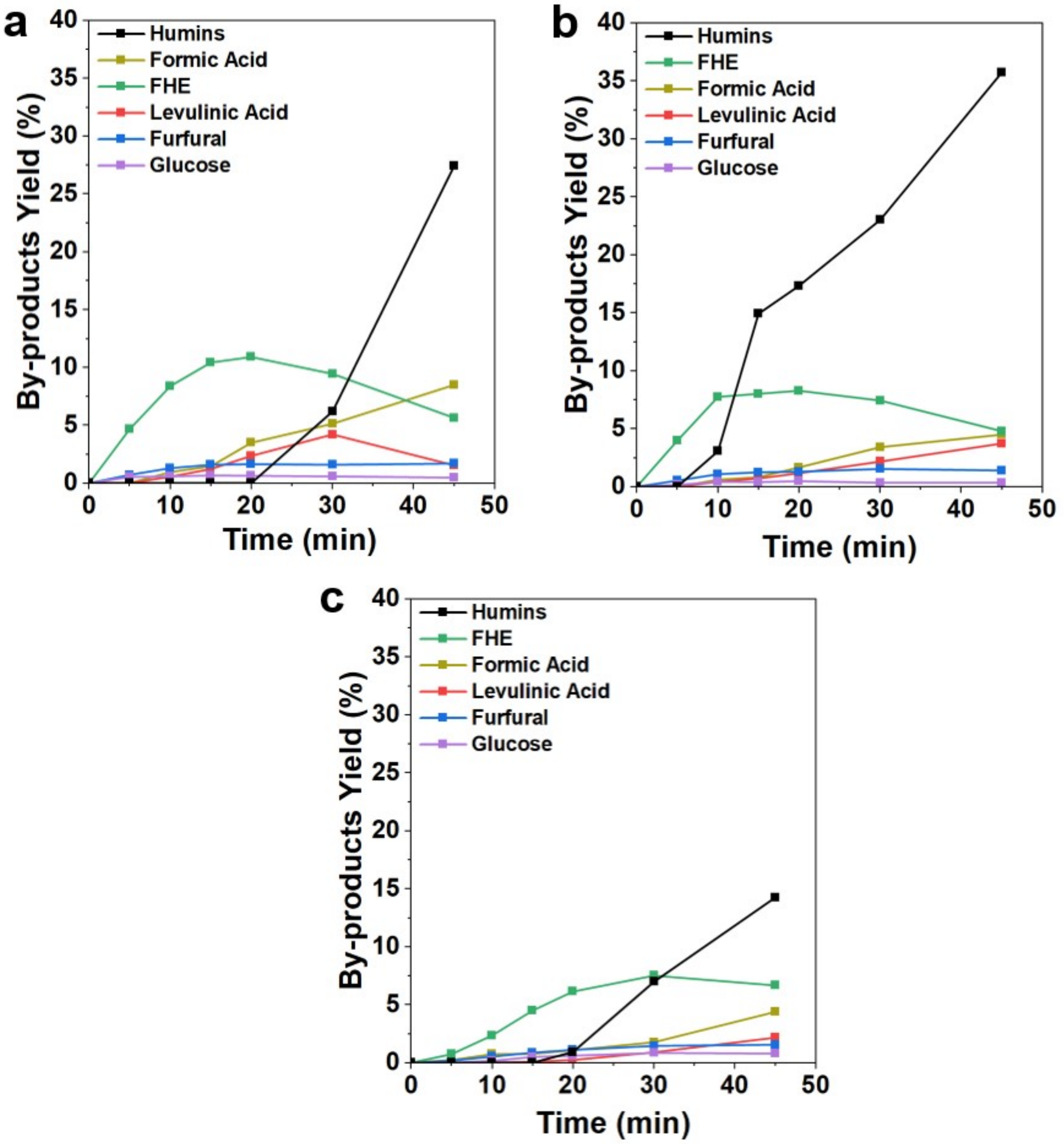

Figure S6. Comparison of by-product yield profiles for aqueous fructose dehydration at 175 °C over (a) RSO_3H/Stellate, (b) RPO_3H_2/Stellate, and (c) RCOOH/Stellate. Reaction conditions: 10 cm^3, 0.28 M aqueous fructose solution, 0.2 g catalyst, 900 rpm, microwave irradiation.

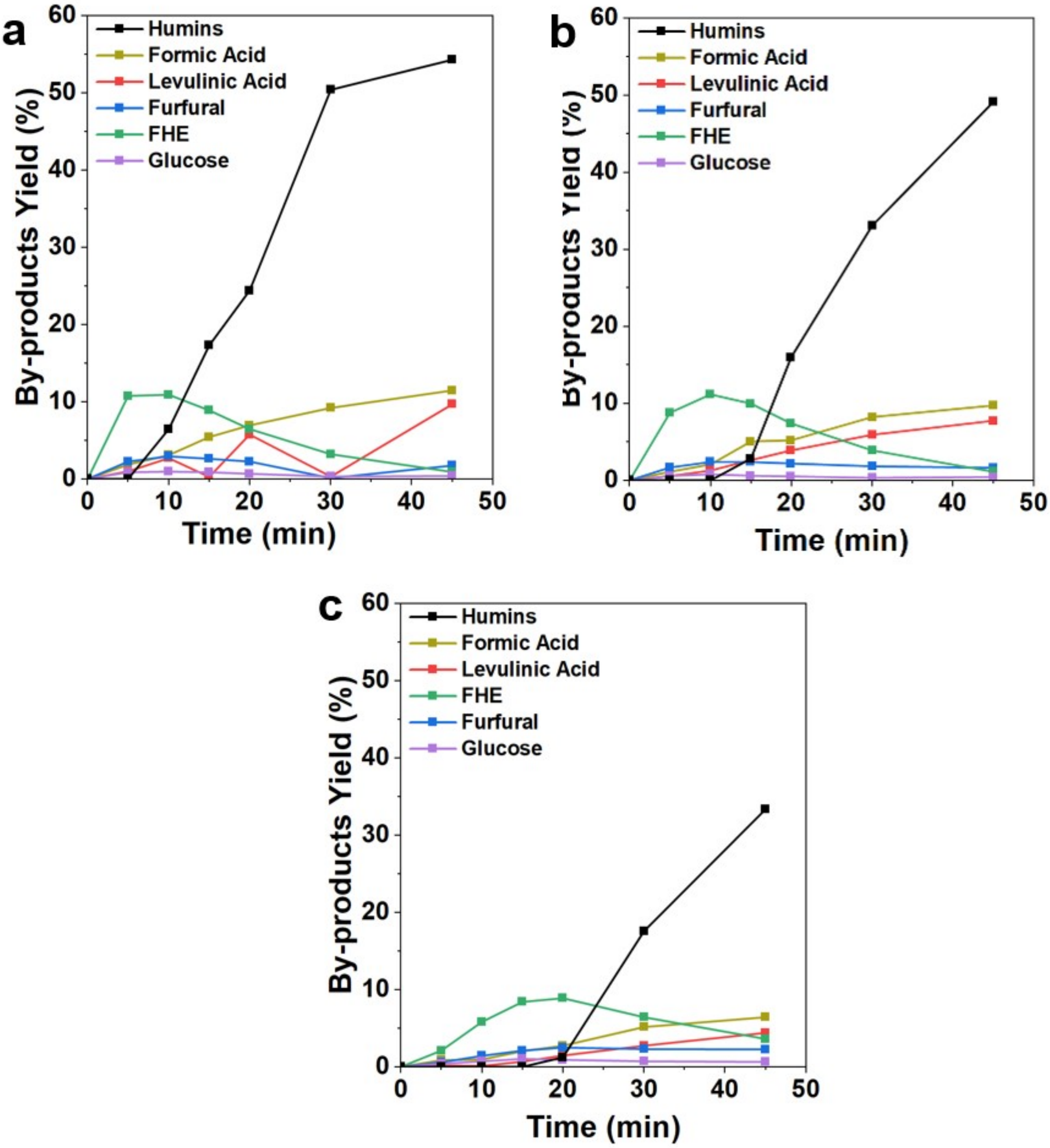

Figure S7. Comparison of by-product yield profiles for aqueous fructose dehydration at 185 °C over (a) RSO_3H/Stellate, (b) RPO_3H_2/Stellate, and (c) RCOOH/Stellate. Reaction conditions: 10 cm^3, 0.28 M aqueous fructose solution, 0.2 g catalyst, 900 rpm, microwave irradiation.

Ta*ble S1*. E-factor (E) (Low), Mass intensity (MI) (low), reaction mass efficiency (RME) (high), and energy economic coefficient (ε) (high) as green metrics of the aqueous fructose dehydration

Catalyst	Temp / ºC	Time / h	HMF yield / %	E-factor (Humins as sole waste)*	E-factor (All by-products as waste)*	MI	RME / %	ε / mmol kJ^{-1}
RSO_3H/Stellate	165	0.75	65.3	0.96 (46.2)	1.19 (46.4)	3.1	45.7	0.0591
RPO_3H_2/Stellate	165	0.75	64.4	0.92 (46.0)	1.22 (46.3)	3.1	45.1	0.0543
RCOOH/Stellate	165	0.75	44.8	2.03 (68.2)	2.19 (68.3)	4.5	31.4	0.0329
RSO_3H/Stellate	175	0.33	69.2	0.77 (42.7)	1.06 (43.0)	2.9	48.4	0.0909
		0.75	53.6	1.35 (55.5)	1.67 (55.8)	3.8	37.5	0.0313
RPO_3H_2/Stellate	175	0.50	57.2	1.23 (51.2)	1.50 (51.5)	3.5	39.9	0.0509
		0.75	48.0	1.63 (60.9)	1.98 (61.3)	4.2	33.6	0.0286
RCOOH/Stellate	175	0.75	63.2	1.00 (47.9)	1.26 (48.2)	3.2	44.2	0.0368
RSO_3H/Stellate	185	0.17	68.1	0.78 (43.4)	1.10 (43.7)	3.0	47.6	0.1549
		0.75	21.0	4.43 (142.9)	5.82 (144.3)	9.6	14.7	0.0106
RPO_3H_2/Stellate	185	0.25	71.6	0.70 (40.5)	0.99 (40.8)	2.8	50.1	0.1108
		0.75	30.2	2.92 (97.3)	3.73 (98.1)	6.7	21.1	0.0156
RCOOH/Stellate	185	0.33	71.8	0.75 (42.1)	0.99 (42.3)	2.8	50.2	0.0842
		0.75	48.5	1.55 (62.6)	1.94 (63.0)	4.2	34.0	0.0253

* values in parentheses reflect calculations in which the solvent is also included in the waste stream

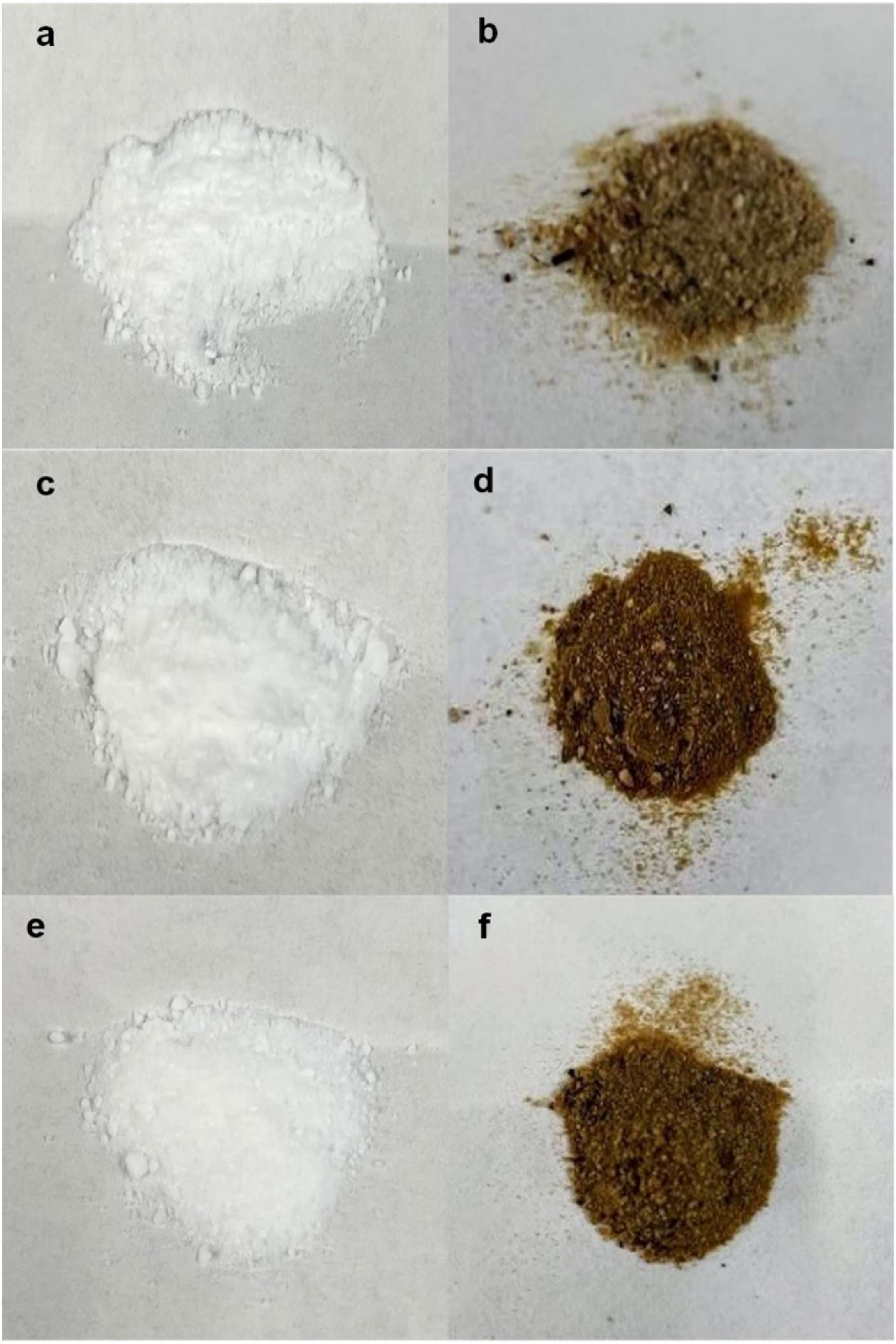

Figure S8. Appearance of RSO_3H/Stellate (a) fresh and (b) spent, RCOOH/Stellate (c) fresh and (d) spent, and RPO_3H_2/Stellate (e) fresh and (f) spent. Spent catalysts are post aqueous fructose dehydration at 165 °C for 45 minutes. All samples were recovered post-reaction by centrifugation and dried at 80°C for 24 h.

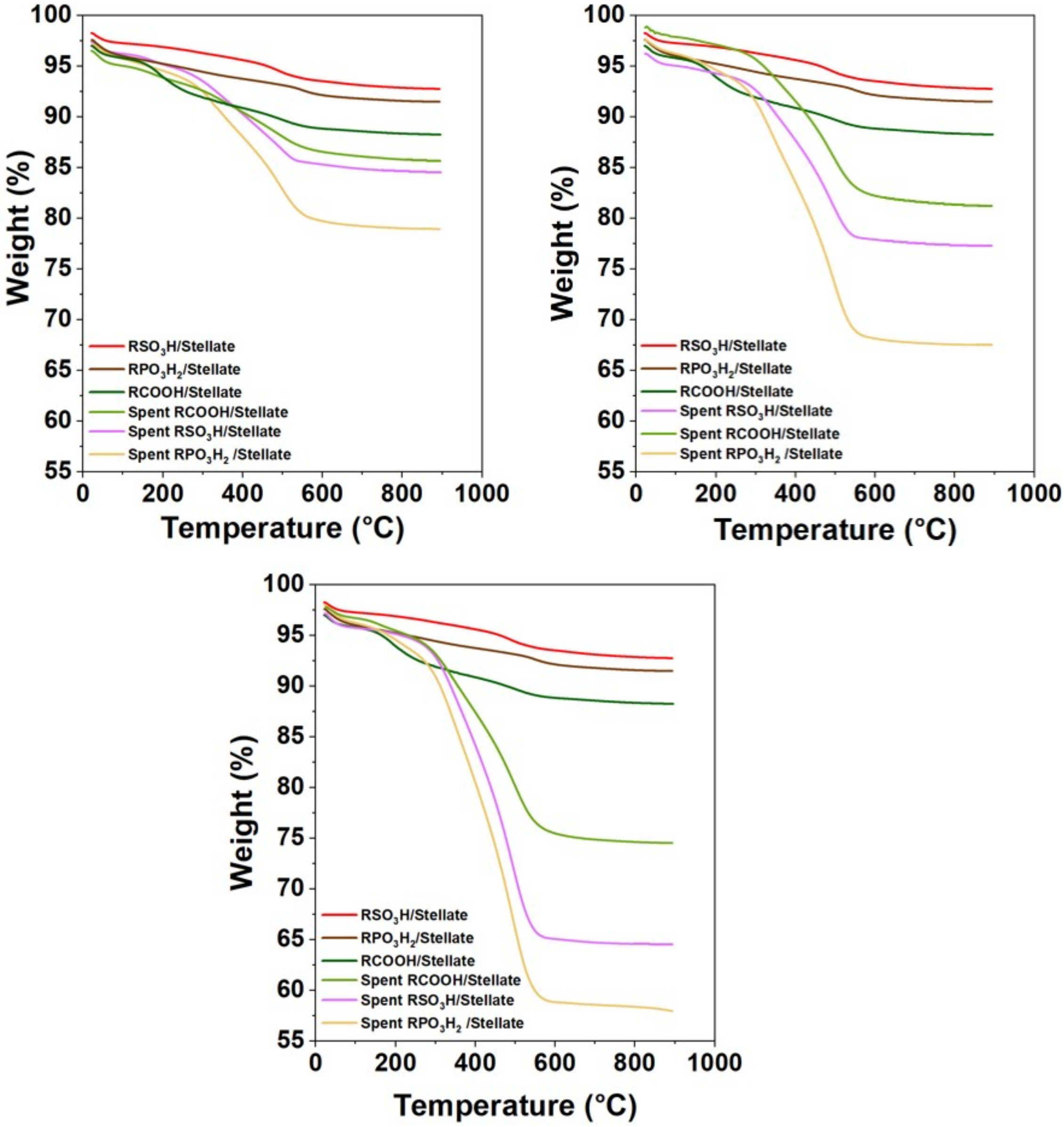

Figure S9. Comparison of TGA curves of fresh and spent catalysts following fructose dehydration at (a) 165 °C, (b) 175 °C, and (c) 185 °C.

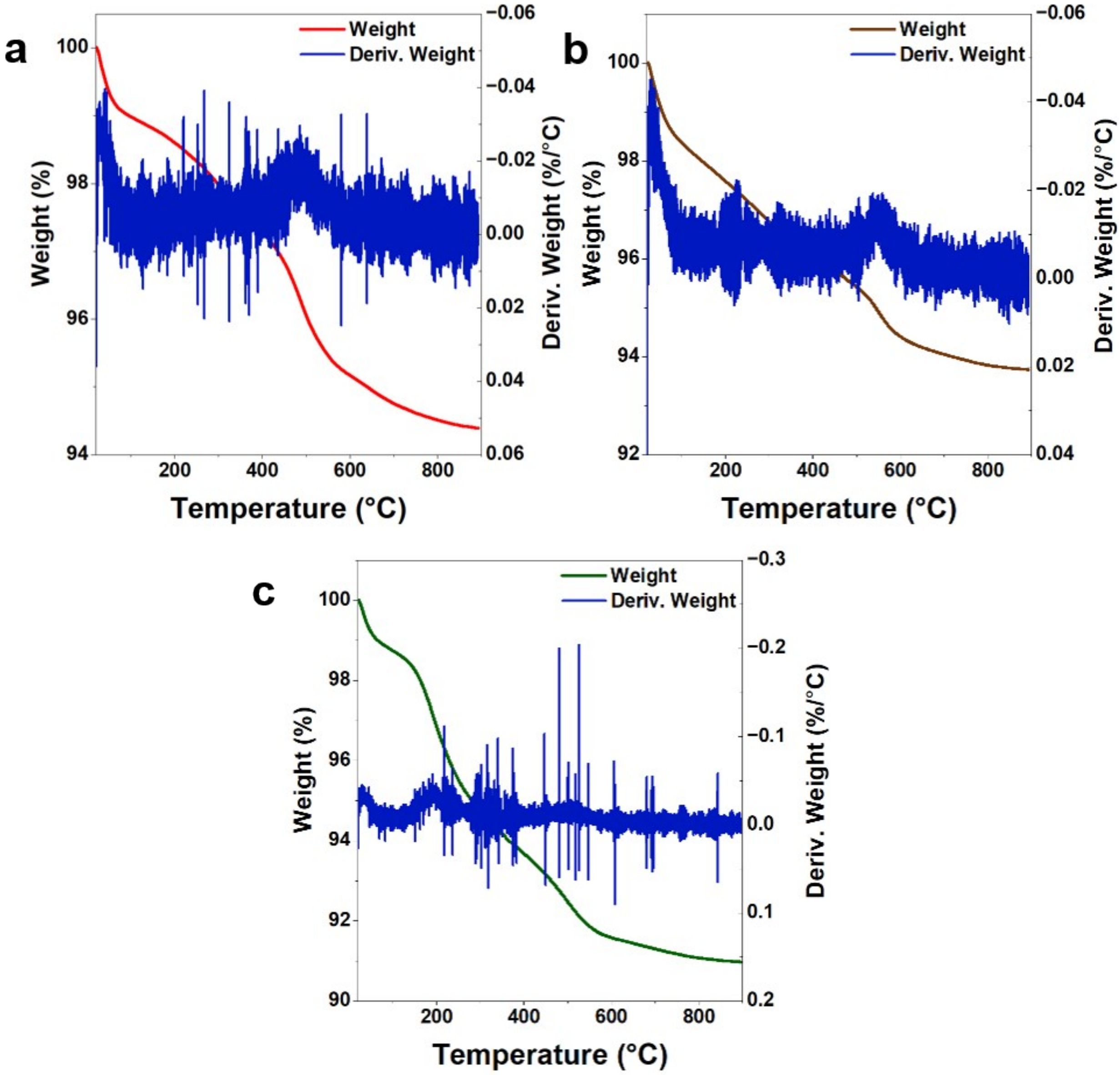

Figure S10. TGA (left axis) and DTG (right axis) curves of fresh (a) RSO_3H/Stellate, (b) RPO_3H_2/Stellate, and (c) RCOOH/Stellate

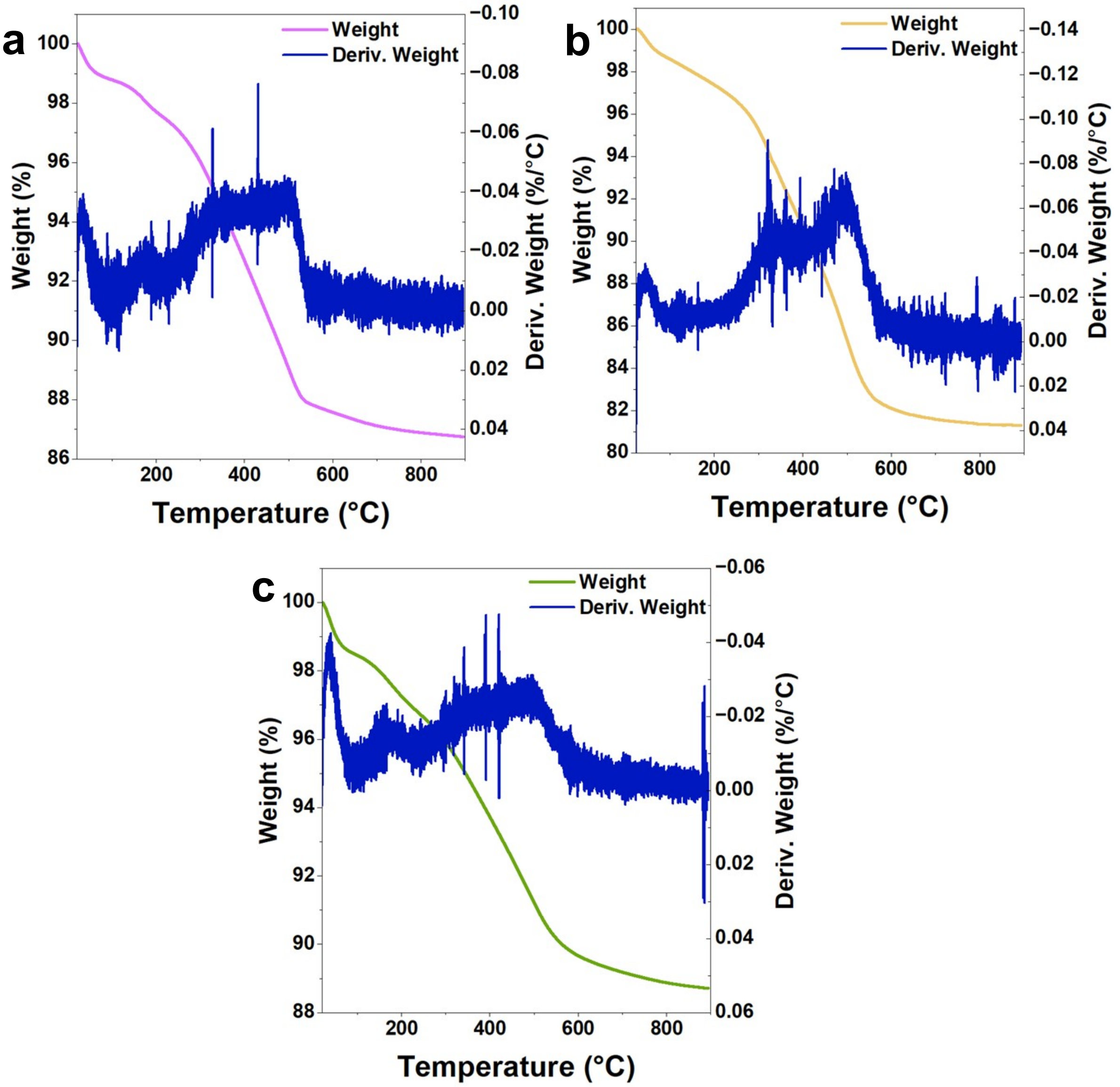

Figure S11. TGA (left axis) and DTG (right axis) curves of spent (a) RSO_3H/Stellate, (b) RPO_3H_2/Stellate, and (c) RCOOH/Stellate following aqueous fructose dehydration at 165 °C.

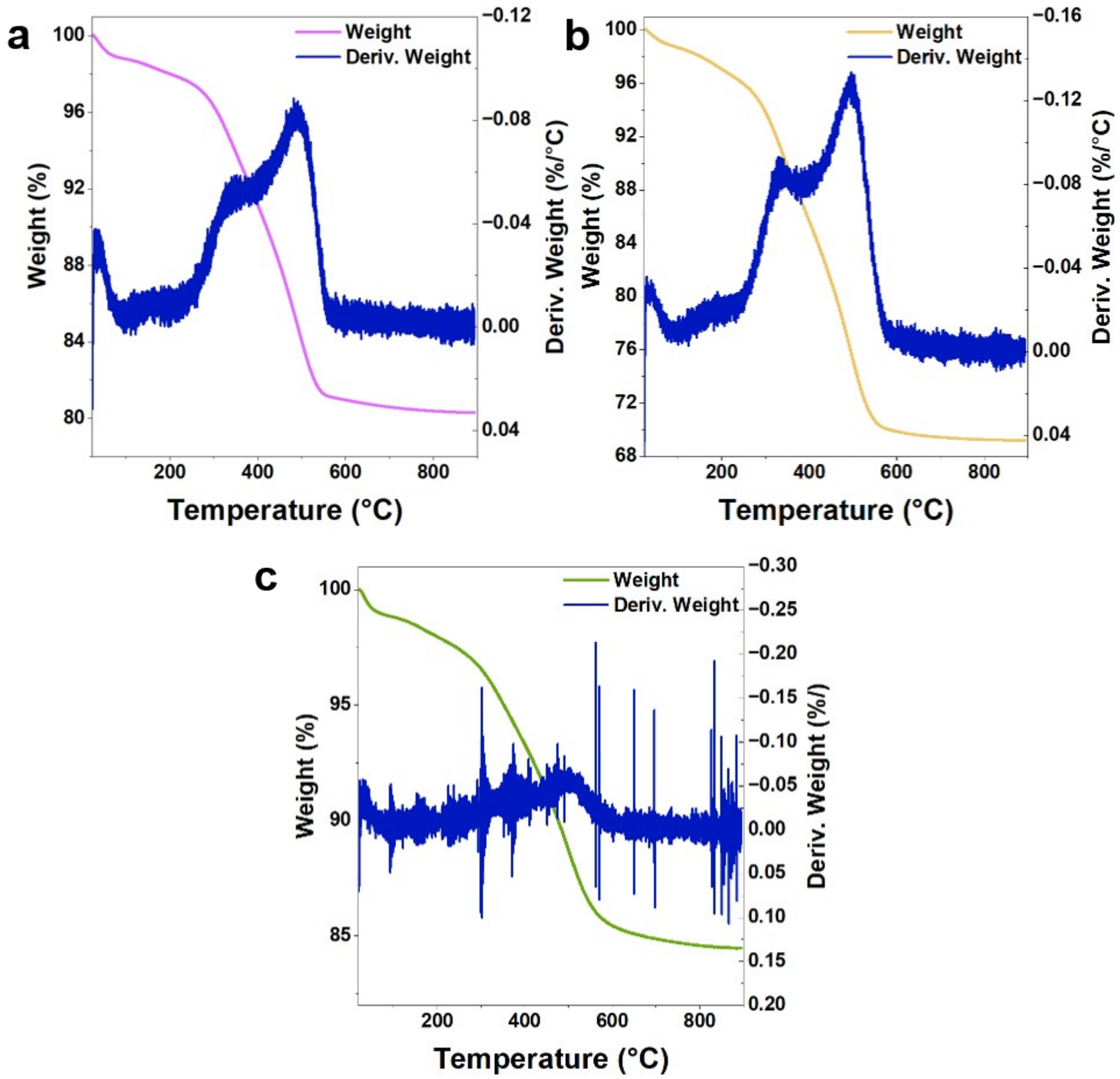

Figure S12. TGA (left axis) and DTG (right axis) curves of spent (a) RSO_3H/Stellate, (b) RPO_3H_2/Stellate, and (c) RCOOH/Stellate following aqueous fructose dehydration at 175 °C.

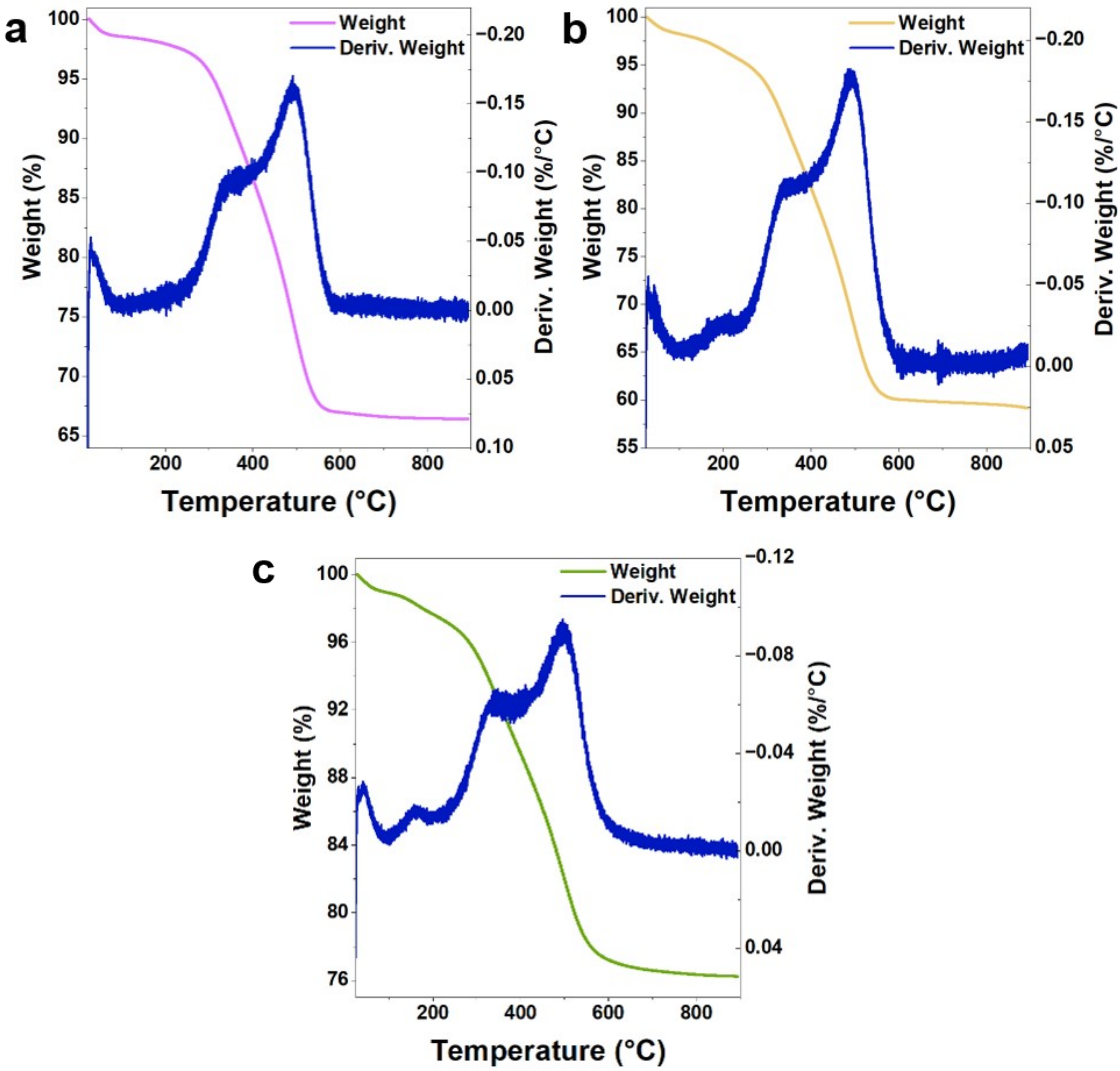

Figure S13. TGA (left axis) and DTG (right axis) curves of spent (a) RSO_3H/Stellate, (b) RPO_3H_2/Stellate, and (c) RCOOH/Stellate following aqueous fructose dehydration at 185 °C.

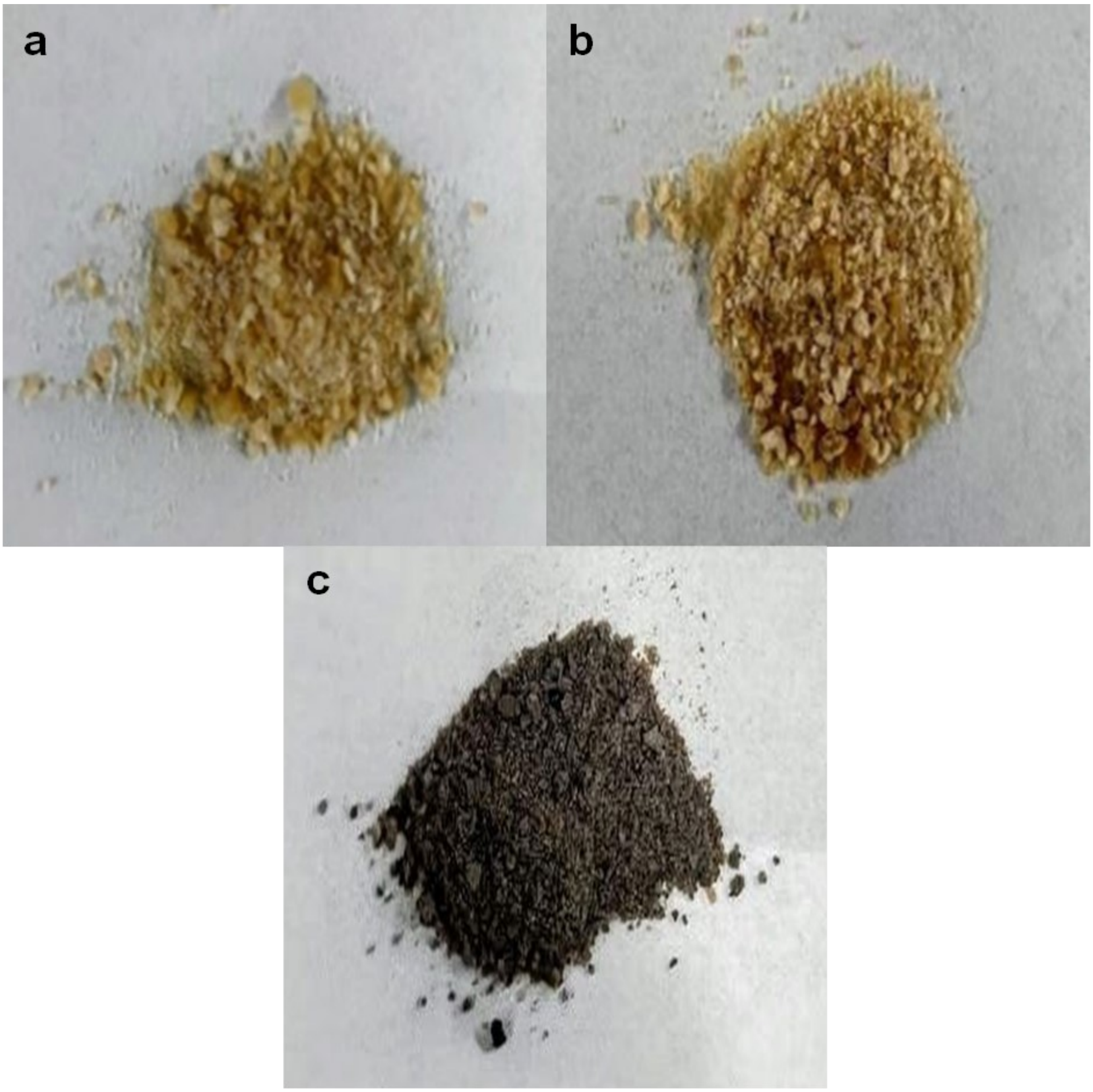

Figure S14. Appearance of regenerated spent (a) RSO_3H/Stellate, (b) RCOOH/Stellate, and (c) RPO_3H_2/Stellate following aqueous fructose dehydration at 165 °C. RSO_3H/Stellate and RCOOH/Stellate were regenerated with H_2O_2, and RPO_3H_2/Stellate was regenerated with HCl.
